# Diving ecology of a pelagic seabird, the Little Auk *Alle alle,* in a changing Arctic revealed by multiyear tracking and optical zooplankton data

**DOI:** 10.1186/s40462-026-00679-1

**Published:** 2026-07-23

**Authors:** Pauline Bodson, Kaja Balazy, Katarzyna Błachowiak-Samołyk, Rafał Boehnke, Lech Marek Iliszko, Kristin Piening, Martyna Syposz, Lech Stempniewicz, Emilia Trudnowska, Katarzyna Wojczulanis-Jakubas, Dariusz Jakubas

**Affiliations:** 1 Department of Vertebrate Ecology and Zoology, Faculty of Biology, University of Gdańsk, Wita Stwosza 59, Gdańsk, PL-80-308 Poland; 2 Institute of Oceanology, Polish Academy of Sciences, Powstańców Warszawy 55, Sopot, PL-81–712 Poland

**Keywords:** Diving strategies, TDR-GPS, LOPC-CTD, Diet, Hydrographic conditions

## Abstract

In a rapidly warming Arctic, seabirds serve as sentinels of ecosystem change, reflecting shifts in oceanographic conditions and prey dynamics. The Little Auk *Alle alle* is a High-Arctic zooplanktivorous seabird that forages mainly on cold-water *Calanus* spp. during the breeding period. To assess its response to high interannual variability in environmental conditions and prey availability, we investigated the foraging ecology of chick-rearing adults breeding in one colony on Spitsbergen over a period of five hydrographically contrasting years. Based on previous studies we considered three potential foraging strategies adopted by Little Auks: optimal (optimal foraging conditions), effort (unfavorable conditions) and reactive (intermediate conditions). To investigate Little Auks foraging behavior we used Temperature-Depth Recorders (TDRs) combined with Global Positioning System (GPS) loggers. To assess prey availability, we used Laser Optical Plankton Counter (LOPC) coupled with a conductivity–temperature–depth (CTD) sensor to collect at-sea high resolution zooplankton distribution data across bird’s feeding grounds. Based on those data we modelled *Calanus* spp. abundance between and within years at different combinations of depth and temperature to show variability of foraging condition within the water column. We characterized diving patterns of Little Auks using a Self-Organizing Map, an artificial neural network, based on 10 TDR-based parameters of dives. We distinguished three diving modes differing in dive duration, depth and shape: short, shallow dives (SSM), intermediate dives (IIU), and deep long dives (DLV). Little Auks adopted two primary strategies: optimal-foraging, dominated by SSM targeting the preferred prey; and reactive-foraging, combining SSM and DLV and supplementing the preferred food with alternative prey. Birds switched between both foraging strategies across years depending on hydrographic conditions which influence the availability of preferred prey. Surprisingly, the optimal-foraging occurred in environmentally contrasted years: in the coldest (2021), the warmest (2023) and the mixed-regime (2024) year. The reactive-foraging prevailed in warm (2018) and mixed-regime (2022) years. In all years, the preferred prey *Calanus glacialis* was supplemented in various proportions by other prey items like *Calanus finmarchicus*, larvae of *Euphausiacea* or larvae of hermit crab *Pagurus* spp. Our study indicates foraging flexibility of Little Auks in a changing High Arctic environment.

**Supplementary Information:**

The online version contains supplementary material available at https://doi.org/10.1186/s40462-026-00679-1.

## Background

In the rapidly warming Arctic, seabirds have emerged as vital sentinels of ecosystem change [1, 2]. Their movements and foraging strategies often reflect shifts in oceanographic conditions and prey availability [3–8]. These avian indicators offer an insight into the health and transformation of marine environments under climate changes. Nowadays, owing to the cutting-edge technological advancements, researchers can track the fine-scale behavior of seabirds using miniaturized data loggers, while simultaneously monitoring their surrounding environment with unprecedented precision. This synergy of biological observation and environmental sensing enables a deeper understanding of how seabirds, and by extension the Arctic ecosystem, are responding to the accelerating pace of climate change.

Building on this technological leap, the development of advanced tracking devices now enables investigation of seabird feeding ecology by disentangling their three-dimensional foraging behaviors, including flying, swimming, and diving. These advances allow researchers to explore ecological dynamics with spatial and behavioral resolution. Global Positioning System (GPS)-tracking devices primarily provide information about the first two components [9, 10], i.e., geographic location (latitude and longitude). Tracking data can then be combined with remote sensed data (e.g., environmental data) to investigate habitat use. The third component, vertical dimension, can be monitored using additional sensors like pressure barometer, altimeter or accelerometer. Capturing the vertical component of movement is vital for understanding predator foraging behaviour, as the energetic costs of vertical displacement can strongly affect hunting strategies and prey acquisition [11–14]. Moreover, many aquatic environments exhibit pronounced vertical stratification in key environmental parameters such as temperature, salinity, and primary productivity, which can significantly influence prey distribution and availability [15–18].

Diving seabirds utilize the three-dimensional foraging space, exploiting different depths of the water column depending on the type, distribution, and behavior of the prey they pursue. This vertical dimension of their foraging strategy is particularly important to understand, as studies continue to demonstrate that variations in dive depth and duration can be an adaptive response to changes in prey and environmental conditions. For example, the Brünnich’s Guillemot *Uria lomvia* adopts U-shaped dives to capture benthic prey and V-shaped dives to capture pelagic prey [19]. Another example is the Northern Gannet *Sula bassana* which has been recorded to perform deeper and longer dives to reach small pelagic fishes, located in deeper layers of the water column, compared to deep but short dives to capture large pelagic fishes [20]. Thus, investigating a seabird’s foraging ecology should include diving profiles, because using solely two-dimensional spatial data (i.e., horizontal location) may introduce bias.

In this study, we investigated the foraging ecology of a small pelagic seabird, the Little Auk (or Dovekie *Alle alle*) in three dimensions using GPS-TDR loggers. This species breeds exclusively in the High Arctic and is the only strictly zooplanktivorous seabird in the North Atlantic [21]. The Little Auk is characterized by extremely high energetic demands compared to other seabirds due to their high wing loading [22, 23] and high costs of diving [14]. Thus, during the breeding season it forages mainly on energy-rich copepods associated with cold Arctic water masses (*Calanus glacialis* and *Calanus hyperboreus*) [24, 25]. The distribution pattern of copepods, and thus their availability to predators, is characterized by high spatio-temporal variability [26, 27]. Consequently, Little Auks face the challenge of effectively identifying and utilizing prey concentration hotspots (patches) that may occur at different depths [28]. In many regions of the European Arctic, cold water *Calanus* copepods are being steadily replaced by warmer-water prey species, most notably by *C. finmarchicus *[29–31]. As *C. finmarchicus* is usually more abundant in waters warmer than 6 °C and is usually considered less rich in lipid (depending on local environmental conditions) [32–35], thus it may represent a less profitable prey for Little Auks especially in areas where Arctic waters predominate [36]. Consequently, depending on the availability of the preferred prey, Little Auks are forced to adjust their foraging ecology, either by performing longer foraging trips, deeper dives, or by collecting alternative prey items [37, 38]. Therefore, the Little Auk’s diet and breeding range make it a perfect sentinel of changes in environmental conditions and zooplankton communities in the High Arctic [21], a region experiencing extreme climate change (e.g., [39]). The Arctic Ocean has been recognized as a hotspot of decadal warming [40–45], affecting both marine and terrestrial ecosystems [46, 47].

We aimed in this study to characterize interannual variability in the diving behavior of Little Auks breeding in a large colony on Spitsbergen, using GPS–TDR data collected over five years under contrasting environmental conditions. Additionally, we complemented the tracking with concurrent at-sea zooplankton screening in foraging areas of Little Auks, employing a laser optical plankton counter (LOPC) coupled with a conductivity–temperature–depth (CTD) sensor. Combining both approaches enabled us to examine the niche overlap between prey, *Calanus* spp. and Little Auks by comparing the depth explored by foraging birds (recorded by TDR logger) and the depth of their prey aggregation (recorded by LOPC-CTD), and by comparing the temperature during foraging (recorded by TDR logger) and the temperature profile in areas with prey aggregations (recorded by LOPC-CTD). To our knowledge, this is the first study integrating data on feeding grounds and dives (GPS-TDR loggers), the at-sea distribution of prey (LOPC-CTD), and diet composition to provide a three-dimensional view of the Little Auks’ foraging behavior. Based on previous studies of the Little Auk’s foraging ecology [25, 38, 48–53], we considered three potential foraging behavior strategies adopted by Little Auks to respond to interannual variability in their foraging environment, based on two axes: environmental conditions in foraging areas via spatial distribution of Little Auks and diet composition:

1. **Optimal-foraging strategy:** the foraging conditions are optimal, with prevalence of cold-water masses in the foraging areas, both in the horizonal and vertical space. Little Auks primarily perform low-energy-cost shallow dives, and consume the preferred cold-water prey item [*C. glacialis* copepodite stage V (hereafter *C. glacialis* CV)].
2. **Effort-foraging strategy:** the foraging conditions are unfavorable with prevalence of warm water masses in the foraging areas, both in the horizonal and vertical space. Little Auks actively search for isolated cold-water refugia where their favorite cold-water prey aggregate, including also prey concentration hotspots at greater depths. As a result, they perform more frequent high-energy-cost deeper dives. Diet is dominated by the preferred cold-water prey.
3. **Reactive-foraging strategy:** the foraging conditions are intermediate, with a mosaic of both cold and warm waters in the foraging areas. Little Auks mainly pursue cold-water patches for feeding during shallow dives. However, deeper dives may occur when encountering warm water at the surface to access their preferred prey in cold-water patches deeper in the water column. Moreover, if the preferred cold-water prey is not available, they may supplement their diet with alternative prey more easily available in the existing conditions.

**Fig. 1:**
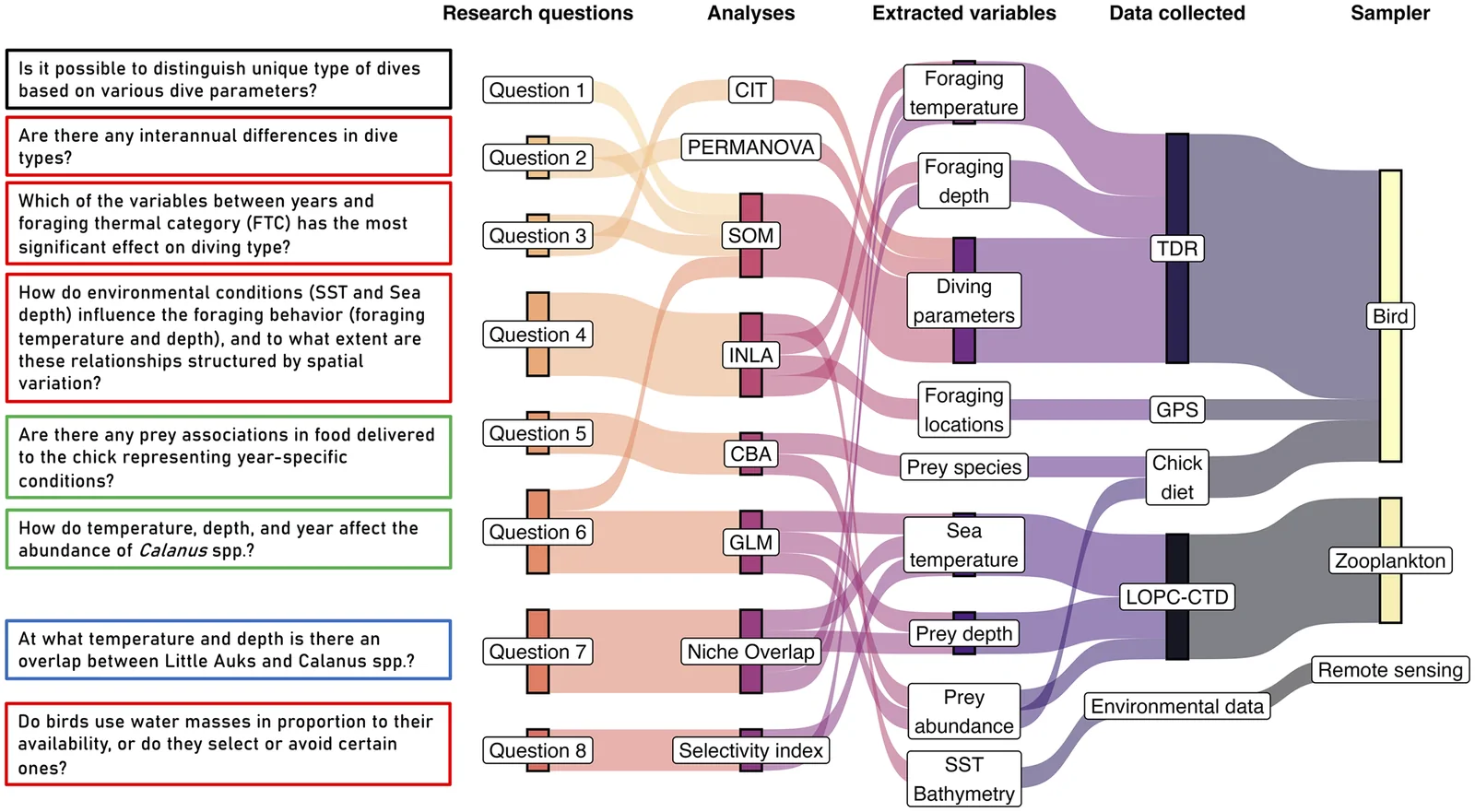
Summary of the research questions presented in this study (thin line boxes on the left). The colors of the boxes indicate which relationships are being explored: relationship between dive type and environmental conditions (red), relationship between prey distribution and environmental conditions (green), relationship between dive type and prey distribution (blue). It also shows type of analyses implemented to answer these questions and data used in the analyses (extracted variables). It shows which equipment or samples were needed to extract the variables (data collected) and the source of the data used to answer the questions (sampler). See abbreviations codes in glossary

To investigate interannual variability in the diving behavior of Little Auks, we formulated a set of eight research questions (Fig. 1) addressing the relationships between dive type and environmental conditions (Question 2, 3, 4, 8), prey distribution and environmental conditions (Question 5, 6) as well as dive type and prey distribution (Question 7). Each question targets specific predictions derived from the three predicted foraging strategies, allowing us to assess which foraging strategies are supported under varying environmental conditions.

## Materials and methods

### Study area

**Fig. 2:**
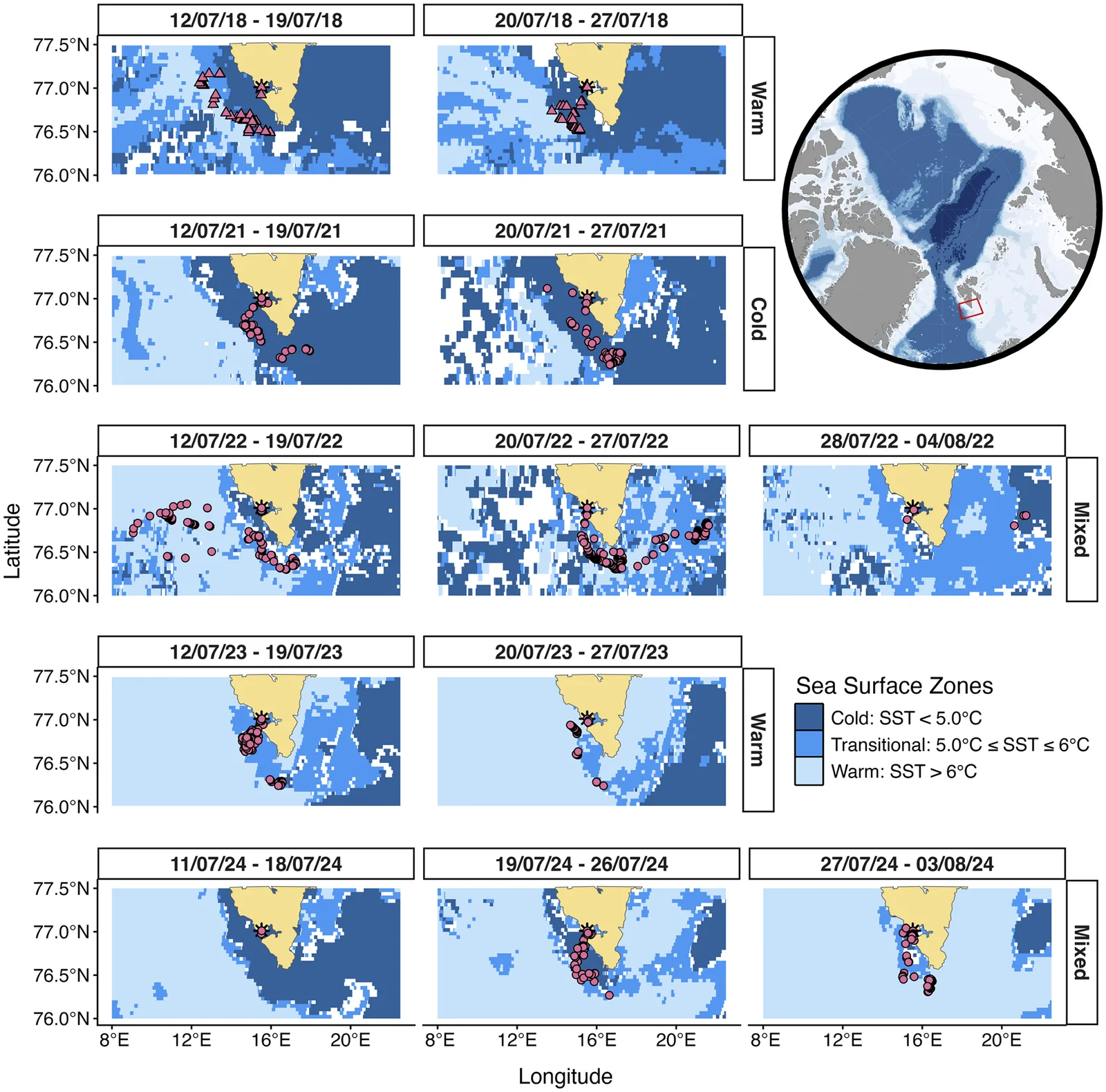
Foraging locations of the GPS-tracked chick-rearing Little Auks (*pink triangles*) breeding in Hornsund, SW Spitsbergen (*black star*) over five years (2018, 2021, 2022, 2023 and 2024). Background: water mass zones based on 8-day mosaics of sea surface temperature (SST) (source: Aqua-MODIS from NASA Ocean Color) during 3 weeks of the chick rearing period of the little auk: cold: SST < 5.1 °C (*dark blue*), transitional: 5.1 °C ≤ SST ≤ 6 °C (*medium blue*), and warm: SST > 6 °C (*light blue*). Inset: location of the studied area in Arctic [background: bathymetry; map generated in the *ggOceanmaps* package [58]]. Classification of the studied years based on CTD data) are provided as side labels. The dates presented for 2024 differ slightly as it was a leap year

We collected our data in the colony at Ariekammen slopes in Hornsund fjord (SW Spitsbergen; 77° 00’ N, 15° 33’E) (Fig. 2), being one of the largest breeding aggregations of Little Auks in Svalbard [54]. The foraging area of birds from the studied colony is extremely dynamic as two water currents interact here, bringing a diversity of prey [53, 55, 56]. The Sørkapp Current brings cold, Arctic-type waters from the northeastern Barents Sea. The Polar Front separates these waters from Atlantic-type waters brought by the West Spitsbergen Current from the Norwegian Sea [57]. The water masses differ among years, affecting the structure of prey communities, and thus the abundance of food for seabirds [7].

### Field work

We conducted our study during five Little Auks chick-rearing periods (2018, 2021, 2022, 2023, 2024). To determine the bird’s foraging area and diving behavior, we deployed loggers with Global Positioning System (GPS) unit combined with a Temperature-Depth-Recorder (TDR) unit (hereafter GPS-TDR loggers). We captured adult birds inside their nests (when they were brooding) and marked them with a stainless-steel band and colored plastic ring for the field identification. We equipped the birds with GPS-TDR loggers manufactured by ECOTONE (Sopot, Poland; size: 27 × 16 × 12 mm, weight 4.2–4.6 g including attachment) from mid to late July (early chick rearing period; Table 1). We attached the loggers to the birds’ dorsal feathers using Tesa tape (code 4965, Tesa Tape Inc. Charlotte, NC, USA). The process of handling and attaching the device took approximately 10 minutes before the birds were released back to their nest. The logger with attachment represented 2.4–2.8% of the birds’ body mass, which complies with the guideline that tracking equipment should not exceed 3% of an individual’s mass [59].

**Table 1:** Summary of data collected with Global Positioning System combined with temperature-depth-recorder (GPS-TDR) logger, and the optical plankton counter (LOPC) coupled with a conductivity–temperature–depth (CTD) sensor

Year	Sample Size (N)				Length of LOPC-CTD transects [km]	Dates of data collection	
	Tracked individuals	Recorded GPS tracks	Recorded dives	LOPC-CTD transects		Birds’ dives with GPS-TDR	At-sea with LOPC-CTD
2018	8	24	3,985	3	39.5–54.5	14–19 Jul	29–30 Jul
2021	15	28	3,905	2	35.7	17–24 Jul	28–29 Jul
2022	8	3	253	2	35.6–47.7	15–19 Jul	03–04 Aug
2023	10	32	5,997	2	15–44	14–21 Jul	01 Aug
2024	15	30	4,749	3	36.4–54.4	18–23 Jul	03 & 05 Aug

In GPS-TDR loggers we programmed the GPS unit to record location every 15 minutes, with activation occurring either 48 hours post-deployment or upon the first contact with seawater. The TDR unit recorded pressure and temperature every 2 seconds using pressure and temperature sensors integrated into the logger casing. A bi-directional radio connection with a GPS-UHF base station located within the colony enabled remote data transfer, eliminating the need to recapture the tagged birds. Data were automatically transmitted to the base station whenever the tagged individuals came within the communication range within a 800 m radius around the antenna.

### Data handling and statistical analyses

We conducted all analyses in R software version 4.5.0 [60].

### Dive analyses

To summarize diving data collected by the TDR unit, we used the *diveMove* package [61]. We excluded very shallow dives (<0.2 m depth), which were probably not associated with foraging (e.g., bathing, wave-induced water splash). The package provided nine dive metrics: ascending time, ascending distance, descending time, descending distance, dive bottom time, dive bottom distance, dive duration, maximal depth, and mean depth at the bottom phase of dive. Because the bottom phase is usually considered as the hunting phase [62], we hereafter refer to the variable as foraging depth. See an example of dive phases together with temperature sensor records in Supplementary Materials Fig. S1.

We computed two more variables. First, the dive shape was defined as the ratio of bottom duration to total dive duration, with higher values indicating more U-shaped dives, and lower values indicating more V-shaped dives [63]. We excluded dives lasting longer than 90 seconds which occurred only in 2018 and represented only 3.5% of all dives recorded (Table 1). Their exclusion improved comaprability with previous studies [64].

Second, we calculated the mean temperature during the bottom phase of each dive. Because this phase occurs between descent and ascent (red points in Supplementary Materials Fig. S1), and corresponds to the hunting phase (chasing and catching prey) [62], we hereafter refer to this variable as foraging temperature. Temperature was recorded every 2 seconds; thus, mean foraging temperature was calculated only for dives with a bottom phase lasting longer than 2 seconds. We excluded temperature values exceeding the maximum temperature recorded across the full water column by the LOPC-CTD in each year, rounded up to the nearest integer degree Celsius (maxTemp_2018_ = 7.9 °C, maxTemp_2021_ = 7.0 °C, maxTemp_2022_ = 9.1 °C, maxTemp_2023_ = 8.9 °C and maxTemp_2024_ = 10.3°C). This procedure removed only 0.07% − 1.05% of records from each annual dataset. For selected analyses (see below), we classified foraging temperature into three foraging thermal categories (FTC) following [37]: **Cold:** (<5.0°C), corresponding to optimal thermal conditions for the preferred prey *C. glacialis *[53, 65]; **Transitional**: (5.0–6.0°C), representing suboptimal conditions for *C. glacialis *[36]; and **Warm:** (>6.0°C), exceeding the physiological threshold of *C. glacialis *[66].

**Fig. 3:**
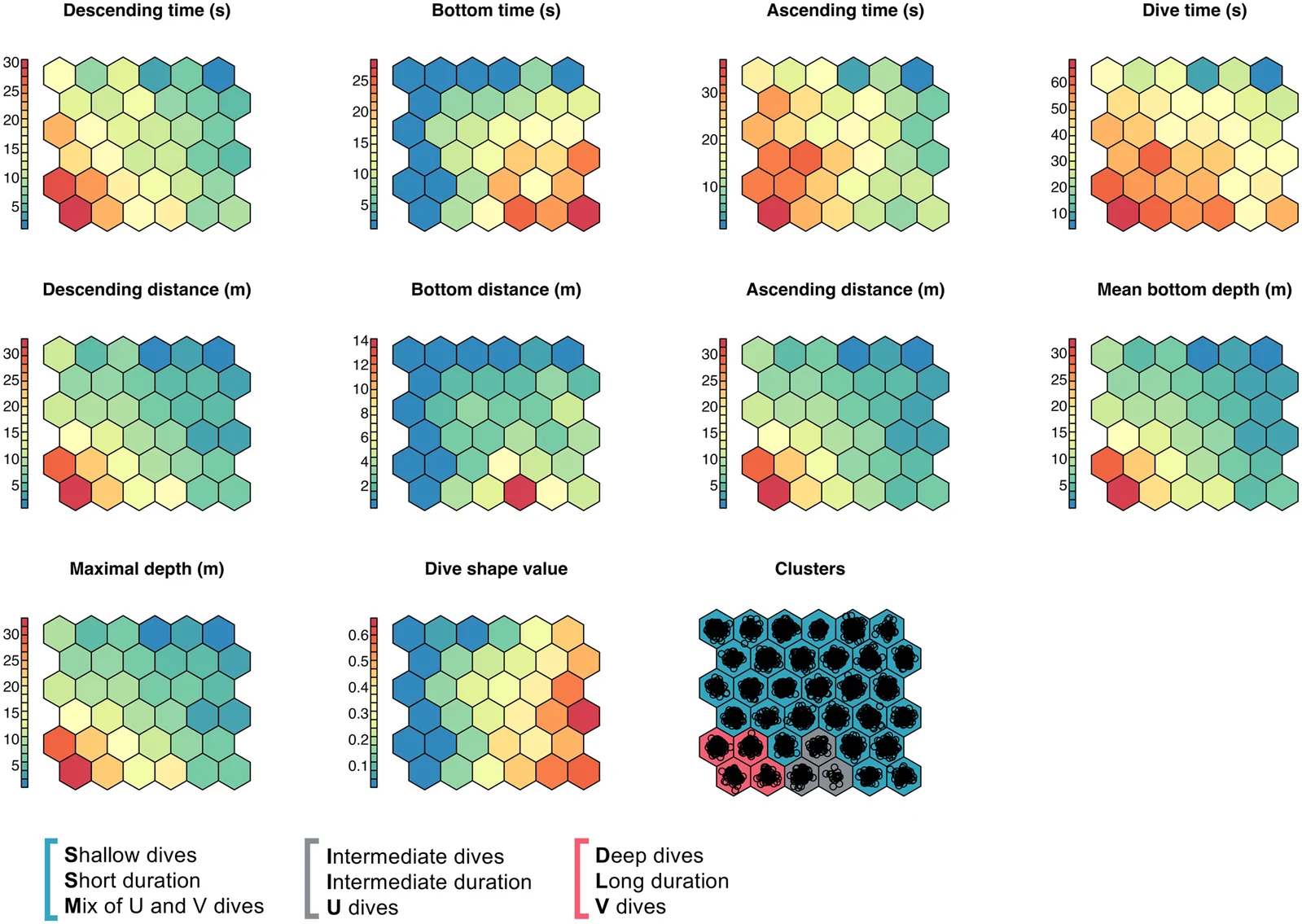
An example of the Self-Organizing map applied to the 10 diving metrics (descending time, bottom time, ascending time, dive time, descending distance, bottom distance, ascending distance, mean bottom depth, maximal depth, dive shape) of the 3,985 dives recorded by the TDR of tracked Little Auks breeding in Hornsund, SW Spitsbergen recorded in 2018. Each dive is classified depending on the value recorded for each diving parameter (i.e., a high value of descending time will be clustered in redder cells and low values of descending time will be clustered in bluer cells). Finally, the model clusters the cells that have a similar pattern into three clusters representing dive types: blue: SSM (shallow dives, short duration, mix of U and V dives), grey: IIU (intermediate dives, intermediate duration, U dives), and red: DLV (deep dives, long duration, V dives)

To characterize diving behavior using multiple TDR-derived metrics, we applied a Self-Organizing Map (SOM), an unsupervised artificial neural network used for dimensionality reduction [67]. We performed analyses using the *kohonen* package [68], based on the 10 dive metrics derived from TDR data collected during the 5 field seasons (Table 1). We used *cutree* and *hclust* functions to split the data into clusters, generating a two-dimensional 6 × 6 hexagonal map (Fig. 3). We evaluated solutions containing 3 to 5 clusters and selected the three-cluster solution because it consistently reproduced the same three dive types (SSM, IIU and, DLV) across all study years.

To investigate variation in four major diving parameters among years and dive type, we used permutational ANOVA from the *permuco* package [69]. We fitted separate models for mean foraging depth, dive duration, dive shape value and mean foraging temperature. We included year, dive type (SSM, IIU and DLV) and their interaction as explanatory variables, while foraging trip identity was treated as stratum. We selected these four response variables because they best describe diving features. When detecting significant effects, we performed pairwise permutational ANOVA to test whether each dive type differed among years (Supplementary Materials Tables S1, S2, S3). To ensure that the pairwise comparisons were not significant due to small deviations in the samples, we ran a Cohen’s d effect size analysis to determine how much SSM differed among years (see Supplementary Materials Table S3).

Finally, we examined how the three SOM-derived dive types were related to environmental conditions using a Conditional Inference Tree (CIT), implemented with the *ctree* function in the *partykit* package [70]. Specifically, we tested whether the proportion of each dive type adopted by individual birds depended on year and foraging thermal category (FTC). To prevent overfitting while maintain interpretability, we constrained the maximum tree depth to three levels.

### Spatial distribution of dives

Since the GPS-TDR logger cannot record the GPS location while birds were submerged, we assigned each dive to the nearest stationary GPS location within a 24-min time window (i.e., up to 12 min before or after the dive). Using this conservative time window, we maximized the spatial accuracy of dive locations retaining a considerable number of dives (N_2018_ = 97, N_2021_ = 4,743, N_2022_ = 323, N_2023_ = 2,440, N_2024_ = 172). We identified stationary locations based on low momentary flight speed, which usually associated with foraging behavior [71]. To determine the speed threshold separating foraging and flight location, we log-transformed the momentary speed separately for each year, and then applied unsupervised model-based clustering using the *mclust* package [72], following [37].

To investigate the spatial variation in diving behavior, we fitted Bayesian hierarchical models using Integrated Nested Laplace Approximation (INLA) implemented in the *R-INLA* package [73]. We modelled spatial autocorrelation using the Stochastic Partial Differential Equation (SPDE) approach with a Matérn Gaussian random field. We fitted two separate Gamma models: one with foraging depth as the response variable and bathymetry (sea depth) as the explanatory variable, and another with foraging temperature as the response variable and sea surface temperature (SST) as the explanatory variable. We included bird identity (BirdID) as a random effect in both models.

We extracted bathymetry (sea depth) and SST for each location using the *extract* function form the *raster* package [74]. We extracted SST from 8-day Level-3 (4-km resolution) AQUA MODIS daytime (11 μm) mosaics [75]. We obtained bathymetry from the GEBCO_2024 model incorporating version 5.0 of the International Bathymetric Chart of the Arctic Ocean (IBCAO), with a spatial resolution of 100 × 100 m [76].

We built the SPDE models using triangular meshes following recommendations of [77]. We chose mesh resolution so that inner triangle edge lengths were between one-fifth and one-tenth of the expected spatial autocorrelation range, while larger outer triangles reduced boundary effects. Because multiple dives shared identical GPS locations, we included in the spatial analyses only years with enough unique locations (N_2021_ = 55; N_2023_ = 45). We set final mesh edges lengths for the inner domain to 3,200 m (2021) and 2,000 m (2023), and 10,000 m and 7,000 m for the outer triangles, respectively.

We specified Penalized Complexity priors for both the spatial range and the marginal standard deviation. We based the prior for the spatial range on the median pairwise distance between GPS locations, representing the expected spatial scale of autocorrelation, whereas we based the prior for the marginal standard deviation on the standard deviation of the corresponding response variable (foraging depth or foraging temperature). We tested several models by adding successively the fix, the random and the spatial effect and chose the final model based on the lowest Deviance Information Criterion (DIC) value (see details in Supplementary Materials Table S4, Fig. S3, Fig. S4).

### Chick diet composition

We captured adult Little Auks feeding chicks at the colony using noose carpets, looped lines and targeted loops. We collected food samples from the birds’ gular pouches with a small spoon within 5–10 min after the capture. We preserved samples in 4% formaldehyde or 96% ethanol (2018–2022) or frozen (2023–2024) and transported to the Institute of Oceanology, Polish Academy of Sciences in Sopot, where prey items were identified. Detailed description of prey items identification is provided in [37].

In total, we collected 110 food samples during the second week of the chick-rearing, with annual sample sizes ranging from 20 to 29 (20 in 2018, 2021 and 2023; 21 in 2022; 29 in 2024). The food sample data used in this study have been partly reported previously [36, 37, 51, 52, 78].

To identify interannual variation in prey associations representing foraging in different microhabitats and environmental conditions, we used Classification Based on Associations (CBA) [79], using the *CBA* function from the *arulesCBA* package [80]. Year was used as the classification variable and raw prey counts as explanatory variables.

We retained five prey categories for the analysis. Based on their habitat preferences, we grouped *C. glacialis* CIV and CV as cold-water prey, while *C. finmarchicus* CIV and CV as warm-water prey [53, 65]. We retained *zoea* larvae of hermit crab *Pagurus* spp. and *furcilia* larvae of *Euphausiacea* sp. in separate categories because their contribution to chick diet has increased over time [37], and we aimed to assess if they represented year-specific dietary composition. We pooled all remaining, less abundant prey items into a single ‘Remaining prey’ category.

CBA identifies recurring prey associations characteristic of particular years and then uses these associations to classify individual food samples. Samples that do not match any identified association are assigned to a default year, defined as the most frequent year among unmatched samples (2022 in this study). As this default classification is not based on prey composition, it was not interpreted ecologically (Table 2).

**Table 2:** Classification rules identified by classification based on association (CBA) describing inter-annual variation in sets of prey items delivered to Little Auks chick by adults. Each rule represents recognized unique year-specific combinations of prey items with their abundances. Numeric range should be interpreted as relative levels (low vs. high) rather than absolute ecological thresholds. Support indicates the proportion of samples that satisfy both the association rule and the specific year. Confidence measures the proportion of the association rule within the year of interest. Coverage represents the proportion of the association rule present in the entire dataset, regardless of year. Samples not matching any prey-based rule were assigned to a default year (2022) and are reported as “Remaining samples” (see details in methods). Inf means infinity

Rule	Year	Association rule	Support	Confidence	Coverage
1	Warm 2018	*C. glacialis* CIV/CV = [1,370, Inf] *C. finmarchicus* CIV/CV = [36.5, Inf] *Pagurus zoea* = [0.5, Inf] Remaining prey = [5.5, Inf]	0.18	0.77	0.24
2	Mixed-Regime 2024	*C. finmarchicus* CIV/CV = [-Inf,36.5) *Pagurus zoea* = [-Inf,0.5)	0.11	0.75	0.15
3	Mixed-Regime 2024	*C. finmarchicus* CIV/CV = [-Inf,36.5) Remaining prey = [5.5, Inf]	0.11	0.71	0.15
4	Cold 2021	*C. glacialis* CIV/CV = [-Inf, 1,370) *C. finmarchicus* CIV/CV = [36.5, Inf] *Pagurus zoea* = [-Inf,0.5) Remaining prey = [5.5, Inf]	0.08	0.69	0.12
5	Warm 2023	*C. finmarchicus* CIV/CV = [-Inf,36.5) *Pagurus zoea* = [0.5, Inf] Remaining prey = [-Inf,5.5)	0.11	0.60	0.18
6	Mixed-Regime 2022	Remaining samples	0.19	0.19	1.00

We described the resulting association rules using three standard metrics: 1) Support indicating the proportion of samples satisfying both the rule and the specific year; 2) Confidence indicating the proportion of the samples assigned to a given year that satisfy the association; 3) Coverage indicating the proportion of the dataset in which the association occurs, regardless of the year [80]. We set the minimum confidence to 0.6 and the minimum support threshold set to 0.1. Raw prey counts were automatically discretized using the minimum description length principle (MDLP) and converted into transactions by the *CBA* function. Candidate classification association rules (CARs) were then generated using the APRIORI algorithm [80].

### LOPC-CTD at-sea sampling

To investigate the high-resolution distribution of zooplankton and environmental conditions, we used a Laser Optical Plankton Counter (LOPC; Brooke Ocean Technology, Canada) coupled with a conductivity–temperature–depth (CTD; SBE 19plus, Sea-Bird Electronics Inc., USA) sensor. The instrument was towed behind the research vessel S/V Oceania in a horizontal undulating trajectory through the upper 60 m of the water column at a constant speed of ~ 4 knots along the transects located within the Little Auk foraging areas (Supplementary Materials Fig. S2). Details of LOPC data processing are described in [81, 82].

We analyzed zooplankton in the 1.0–2.5 mm equivalent spherical diameter (ESD) size fraction, representing mainly the size range of older life stages (CIV and CV) of *Calanus* spp. [82–84]. Thus, size range includes *C. glacialis* CV, the preferred prey item of the Litte Auk [48, 85]. We excluded LOPC data below 45 m because the deepest dive recorded in this study reached 43 m.

We retained the full temperature range recorded by the CTD for two main reasons. First, both *C. glacialis* and *C. finmarchicus* occur in waters colder than 6 °C, while *C. finmarchicus* also occur in warmer waters [36, 51, 65, 86, 87]. Second, Little Auks in the present study foraged in waters with temperature reaching up to 10 °C, justifying the inclusion of higher temperatures in the analysis.

To investigate factors influencing *Calanus* spp. abundance and vertical distribution assessed by the LOPC-CTD, we fitted zero-inflated negative binomial generalized linear models (GLM) using the *glmmTMB* function from the *glmmTMB* package [88]. We retained zero counts to identify areas of low importance for *Calanu*s spp. and, consequently, for Little Auks foraging. We modelled zero-inflation as a function of depth and temperature, allowing the probability of structural zeros (excess zeros beyond those expected from the count distribution) to vary with these environmental variables. We included only CTD-derived depth and temperature as predictors as equivalent variables were available from the TDR data describing Little Auk dives, facilitating direct comparisons between prey and predator distribution. Moreover, both variables have been recognized as most successful in differentiating Arctic and Atlantic zooplankton communities in the Hornsund area [53].

We included depth and sea temperature as continuous predictors and year as categorical predictor. We tested several models with different combinations of main effects and interaction terms and chose the final model based on the lowest Akaike Information Criterion (AIC) value (Supplementary Materials Table S5) using the *AIC* function from the *stats* package [60]. The selected model included interaction of year × sea temperature, depth ×year, and the three-way interaction year × sea temperature ×depth. We performed pairwise comparisons of interaction slopes using the *emtrends* function from the *emmeans* package [89].

Finally, to assess zooplankton abundance within the Little Auk foraging habitat, we used the fitted model to predict *Calanus* spp. abundance across combinations of sea temperatures and depths using the *ggpredict* function from the *ggeffects* package [90]. Predictions were generated for the mean foraging depths of each dive type (SSM, IIU, and DLV) in each study year (2018–2024), across temperature ranging from 0 and 8 °C, reflecting the CTD range of *Calanus* spp. occurrence.

### Classification of studied years and birds’ environmental preference

**Fig. 4:**
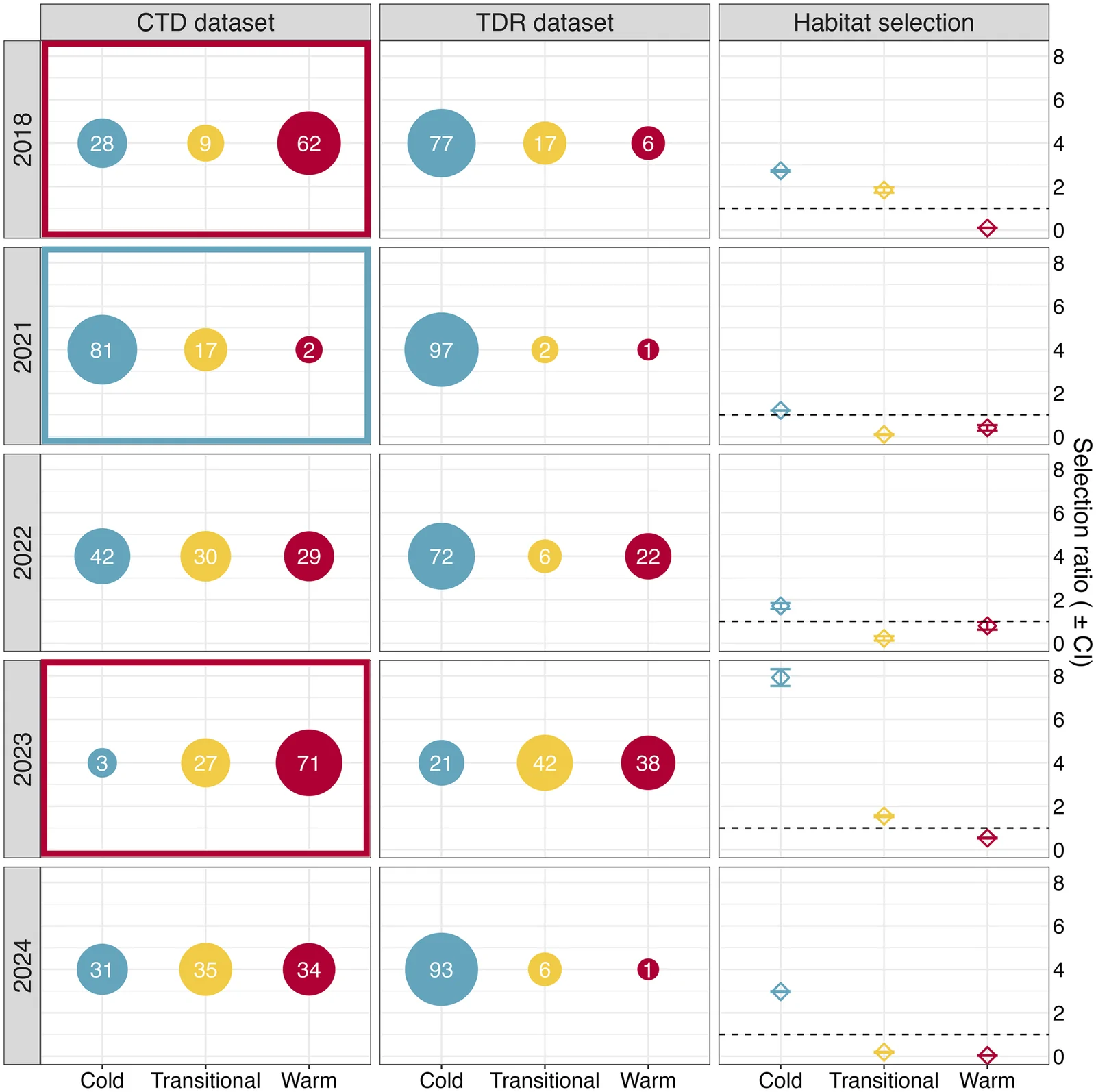
Percentages of data points recorded by CTD and TDR sensors in cold, transitional, and warm waters on the foraging grounds of Little Auks breeding in Hornsund, SW Spitsbergen. The left panel represents the temperature recorded by the conductivity, temperature, and depth (CTD) unit combined with laser optical plankton counter (LOPC) in the transects located in Little Auk foraging areas (see locations of LOPC-CTD transects in Fig. S2, S6). Only patches with more than 450 copepods · m^−3^ of *Calanus* spp. were included in this analysis as lower values might not trigger the foraging behavior of Little Auks. The middle panel presents the temperature recorded by TDR logger, at the bottom phase of the dive (foraging phase) of the studied Little Auks. The right panel shows the selection ratio of foraging habitat by Little Auks. It compares the usage of water patches by Little Auks recorded by the TDR to the available water patches on the foraging grounds recorded by the LOPC-CTD. If the diamond and its confidence interval (CI) are not crossing the dashed line, then it demonstrates a significant preference (values above the line) or avoidance (values below the line) for specific water masses. Otherwise, it means that Little Auks did not preferentially select the water masses. The left panel shows the main foraging thermal category (FTC) displayed as the colored frames around each box: warm (red), cold (blue); the lack of frame indicates mixed regime without prevalence of any FTC

We classified years based on the at-sea temperature measured by the LOPC-CTD (Fig. 4). Consequently, we ran a chi-squared test for goodness of fit from the *stats* package [60] on the proportion of Foraging Thermal Category (FTC) measured separately for each year, with an expected frequency of 1/3 for each FTC representing equal contribution of the different water regimes. The main FTC was selected as the prevalent zone when significant difference from equal proportion of FTC was recorded and standardised residuals were greater than 2 as a rule of thumb.

We investigated the preference of Little Auks for particular FTCs by calculating the selection ratio based on the proportion of the FTC recorded by TDR (representing actual utilized range of temperatures) and CTD (representing range of temperatures available in the foraging areas). We calculated the habitat selection ratio and its 95% confidence intervals (CI) using the Agresti-Coull-adjusted method. Values of the ratio with CI entirely above 1 indicated preference for a given FTC when those entirely below 1 indicated avoidance; index values with CI crossing 1 indicated neutrality. We computed the index with the *ci.prat.ak* function from the the *asbio* package [91].

### Overlap between Little Auk diving and *Calanus* spp. occurrence niches

Since the home ranges of the tracked birds overlapped spatially with the LOPC-CTD transects (Supplementary Materials Fig. S2), we compared Little Auk diving behavior (depth and temperature) with the depth and temperature structure of *Calanus* spp. aggregations recorded by the LOPC-CTD and loggers.

Due to logistical constraints, we were not able to collect LOPC-CTD and GPS-TDR data simultaneously. The temporal mismatch between datasets ranged from 4 to 14 days among years (Table 1). We assumed that spatial distribution of *Calanus* spp. aggregations remained broadly similar over late July, as water masses in this part region are largely structured by bottom topography, which stabilizes the position of the Polar Front on the shelf break [92].

We characterized the environmental niche of *Calanus* spp. using temperature and depth data derived from the CTD integrated with LOPC, and the diving habitat of Little Auks using foraging depth and temperature from the GPS–TDR records. Niche breadth for both *Calanus* spp. presence and Little Auk foraging habitat was estimated using the *nicheROVER* package [93]. For each variable, we generated 1,000 posterior draws assuming a probability level of alpha 0.95. For visualization, we randomly selected 10 projections of the resulting distribution to construct two-dimensional niche representations (Supplementary Materials Fig. S5).

We quantified the probability of overlap between *Calanus* spp. and Little Auks foraging habitat in two ways. First, we used the *overlap* function from the *nicheROVER* package to estimate the probability that Little Auk foraging occurred within the environmental niche of *Calanus* spp. A high overlap value indicated that the birds were able to exploit *Calanus* spp. patches, whereas a low overlap value indicated that the birds had difficulty finding and exploiting *Calanus* spp. patches. Second, we estimated total niche overlap using the *maxLikOverlap* function from the *SIBER* package [94]. In this analysis, we restricted *Calanus* spp. data to abundance values higher than 450 copepods · m^−3^, corresponding to the lowest prey density previously reported to be exploited by Little Auks [95].

## Results

### Classification of studied years and birds’ environmental preference

The environmental conditions recorded by CTD in foraging areas of Little Auks lead us to classify 2018 and 2023 as warm regime years (χ^2^ test, all *p* < 0.001), 2021 as a cold regime year (χ^2^ test, *p* < 0.001), and 2022 and 2024 as mixed-regime years (χ^2^ test, 2022 *p* = 0.21 and 2024 *p* = 0.88) with almost equal contribution of particular FTC in CTD data.

For the majority of the years studied, Little Auks preferred cold waters for foraging with over 70% of their dives occurring in cold waters (TDR dataset: 77% in 2018, 97% in 2021, 72% in 2022 and 93% in 2024). Even during warm 2023, where only 3% of cold water patches were available, the selectivity index showed clear selection for cold waters as birds still performed 21% of their dives in cold waters. In the least favorable year 2018 and 2023 they also exhibited preference for transitional waters over warm waters. They avoided warm waters in each studied year (Fig. 4).

### Main dive types

Self-Organizing Map (SOM) classified dives of chick-rearing Little Auks into three clusters representing three dive types differing in diving depth, dive duration, and dive shape. The first dive type is characterized by **S**hallow dives with **S**hort diving duration and the dive shape values classified as a **M**ix of U-shaped and V-shaped dives (hereafter **SSM**). The second dive type consists of dives of **I**ntermediate depth with **I**ntermediate diving duration and with the dive shape characterized mostly by **U**-shaped dives (hereafter **IIU**). The third dive type is described by **D**eeper dives of **L**ong duration and are mostly characterized by **V**-shaped dives (hereafter **DLV**). All three dive types were detected each year, thus, in further analysis, we were able to present and analyze year-specific SOMs results (see example of classification in Fig. 3).

### Dive metrics across years and dive types

**Fig. 5:**
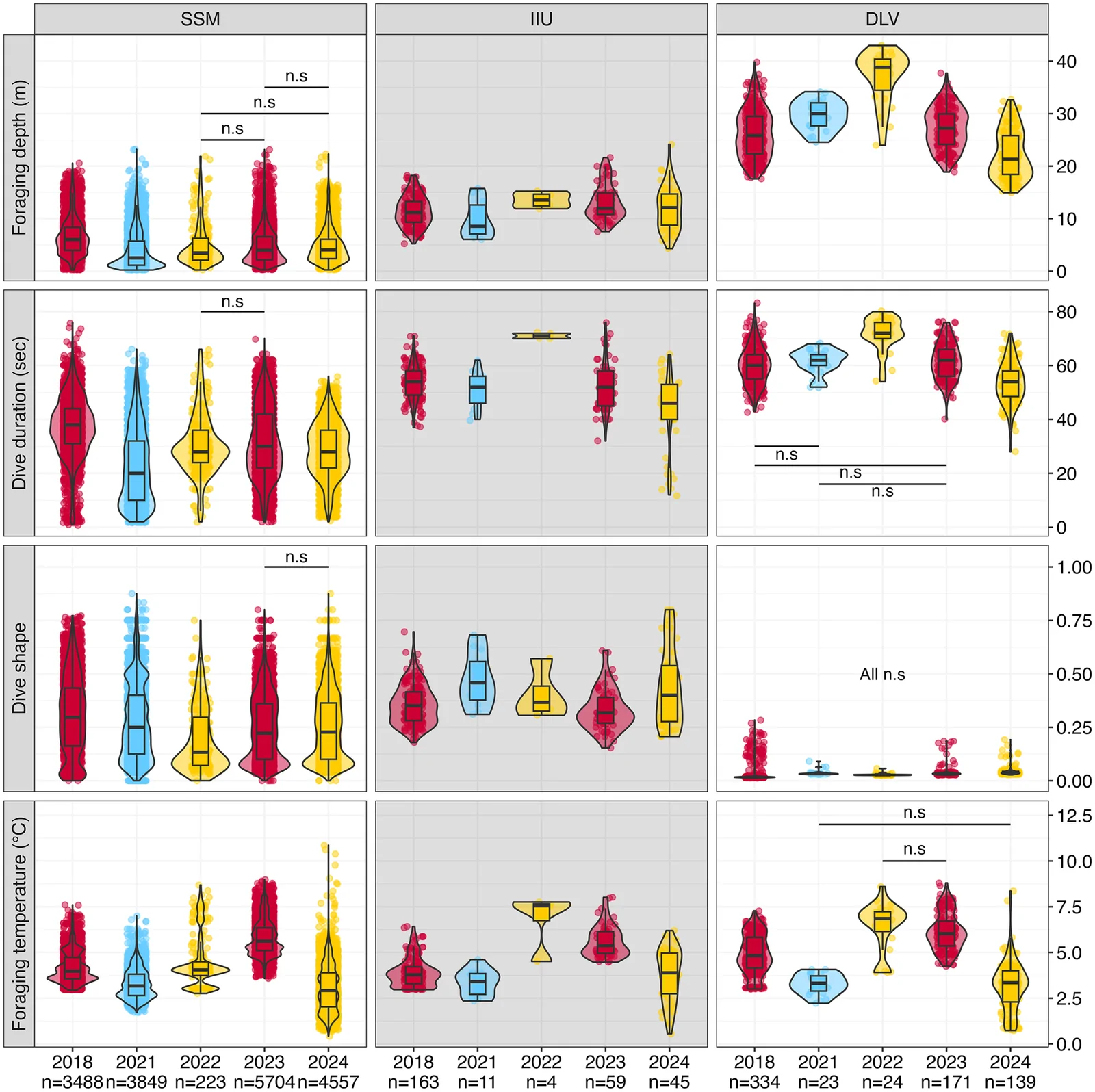
Boxplot of the main diving metrics: foraging depth (m), dive duration (sec), dive shape, and foraging temperature (°C) for each dive type: shallow dives, short duration, mix dive shape (SSM), intermediate depth, intermediate duration, U-shaped dives (IIU) and deep dives, long duration, V-shaped dives (DLV) among the 5 years of study. Lack of significant differences (PERMANOVA, *p* > 0.05) within a given dive type depicted with n.s. bar. Estimates based on fewer than 20 dives should be interpreted with caution. Therefore, the IIU dives (shown on grey background) PERMANOVA results are not shown (they are presented in Supplementary material table S2). Colors represent the main foraging thermal category (FTC) in particular years: red – warm, yellow – mixed-regime, blue – cold. Boxplots show the median (band inside the box), the first (25%) and third (75%) quartile (box), the lowest and the highest values within 1.5 interquartile range (whiskers) and outliers (circles). Violin plot represents probability density

The dive metrics (mean foraging depth, dive duration, and dive shape) and mean foraging temperature differed significantly among years within the same dive type (PERMANOVA, resampled *p* < 0.0002) (Fig. 5, Supplementary Materials Tables S1, S2, S3). Dive duration in SSM dives was longest in warm 2018 and mixed-regime 2022 and shortest in cold 2021, with intermediate values in the other years. Foraging depth followed a similar pattern, with shallower dives in cold 2021 and deeper dives in warm 2018 and mixed-regime 2022, while depths were intermediate in warm 2023 and mixed-regime 2024. Dive shape for SSM dives declined between 2018 and 2022 but increased in 2023 and 2024. Foraging temperature during SSM dives also varied significantly between years, with higher temperature recorded in 2023 compared to all other years.

When looking at DLV dives, the dive duration peaked in mixed-regime 2022, coinciding with the deepest foraging depth observed during the studied years, while shorter and shallower dives were recorded in 2018, 2023 and particularly in 2024. The proportion of time spent at the bottom phase of the dive (i.e., the dive shape) during DLV dives remained low across all years and did not show significant interannual variation. Conversely, foraging temperature varied among years, with higher temperatures recorded in mixed-regime 2022 and warm 2023 compared to warm 2018, cold 2021 and mixed-regime 2024.

IIU dives were rare (1.5% of all dives recorded) and exhibited interannual variability in dive parameters; however, interpretation is limited due to low and uneven sample size. SSM dives, on the other hand, were recorded at a high frequency. Cohen’s effect size analysis revealed that comparisons identified as statistically significant exhibited moderate to very large effect size (see Supplementary Materials Table S3).

### Factors affecting the proportion of dive types

**Fig. 6:**
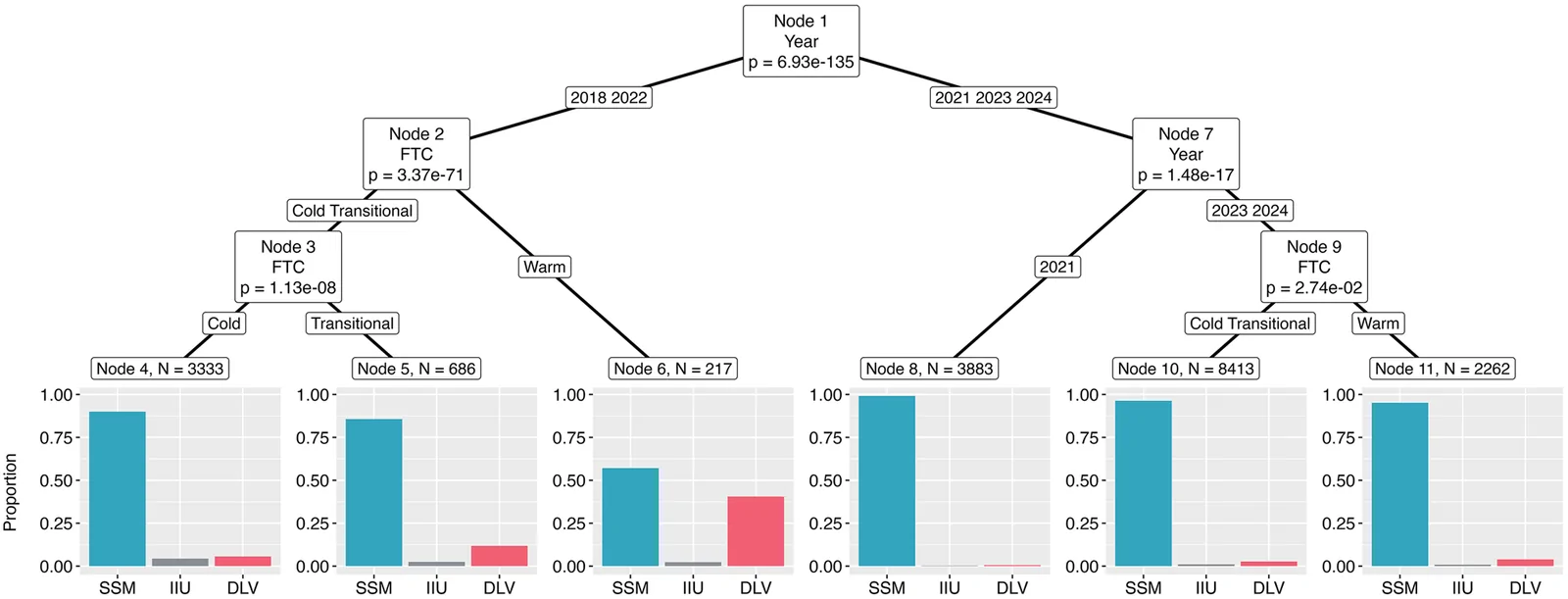
Conditional Inference Tree classifying which variables between the years (2018, 2021, 2022, 2023 and 2024) and foraging thermal categories FTC (cold, transitional, warm) affected the dive type (SSM, IIU or DLV) choice by TDR-tracked chick-rearing Little Auks breeding in Hornsund (SW Spitsbergen). The *p* values present at each node, all highly significant, represent the test of independence between the explanatory variables (year and FTC) and the response variable (proportion of dive type). The enclosed variables have the strongest relationship with the response variable and best describe the difference in usage of dive type. The histogram bars in the terminal node represent the percentage of dive types (SSM, IIU and DLV)

In all studied years SSM represented the most common dive type making up from 61.8% to 100.0% of the dives. The IIU dive type was the least frequently used accounting for 0.3% to 5.4% of the dives, which was preceded by DLV dives accounting for 0.6% − 43.2% of the dives (Fig. 6, Supplementary Materials Table S6).

The Conditional Inference Tree revealed that the proportion of each dive type is affected significantly by the year and the Foraging Thermal Categories (FTC). The proportion of the various dive types used by Little Auks in 2018 and 2022 (Node 1) was significantly different and more variable than in 2021, 2023 and 2024, when usage of SSM was the highest (Fig. 6). The Little Auks’ dive type in 2018 and 2022 depended on the FTC (Node 2), with a higher rate of DLV dives in warm waters (37.9%; Node 6), an almost equal proportion of IIU (5.1%) and DLV (8.1%) dives in cold waters (Node 4), and an intermediate level of DLV dives in transitional waters (12.5%; Node 5), while foraging. In cold 2021, Little Auks dive type was different from all the other years (Node 8), with the SSM dives being almost the only type used regardless the FTC encountered. In 2023 and 2024 (Node 9), the proportion of particular dive types used by Little Auks depended on FTC. The number of DLV was lower in cold (2.6%) and transitional (2.3%) FTC (Node 10) than in warm FTC (4.5%; Node 11) (Fig. 6)

### Spatial distribution of dives

For both 2021 and 2023, the best-supported INLA-SPDE models investigating the effect of sea surface temperature (SST) on foraging temperature, and the effect of bathymetry (sea depth) on foraging depth (models mD_4_2021_, mD_4_2023_, mT_4_2021_ and mT_4_2023_ in Supplementary Materials Table S4) included only a spatial random effect, with no additional support for including individual identity once spatial structure was accounted for (ΔDIC < 4 in all cases; given that ΔDIC values < 7 indicate no clear support for one model over another, we retained the simpler model including only the spatial random effect, following the principle of parsimony [96]). In 2021, the posterior distribution for SST indicated a positive effect (mT_4_2021_ – mean estimate = 0.22, 95% Credible Intervals CI: 0.01–0.44), supporting the association between SST and foraging temperature. Conversely, such a direct effect of SST on foraging temperature was detected neither in 2023 (mT_4_2023_ – mean estimate = 0.05, 95% CI: −0.06–0.16), nor was there any evidence of an effect of bathymetry on foraging depth (mD_4_2021_ – mean estimate = −0.001, 95% CI: −0.006–0.004; mD_4_2023_ – mean estimate = 0.001, 95% CI: −0.003–0.004).

Both foraging parameters showed clear spatial structuring. Foraging temperature was spatially autocorrelated over scales of 2.7–8.2 km in 2021 (mT_4_2021_) and 0.8–2.6 km in 2023 (mF_4_2023_), while foraging depth was autocorrelated over spatial scales of 3.8–11.0 km in 2021 (mD_4_2021_) and 1.7–7.1 km in 2023 (mD_4_2023_). This indicates that dives performed within close proximity were more similar to one another than dives performed further away (Supplementary Materials Figs S3 and S4).

### Prey associations in chick diet

The prey association analysis performed using the Classification Based on Association Rules (CBA) algorithm revealed some year-specific associations of delivered prey (Table 2). Both warm 2018 and cold 2021 were characterized by high abundance of *C. glacialis* CIV/CV in detected prey associations. However, these two years also shared high abundance of *C. finmarchicus* CIV/CV and Remaining prey item in delivered food. Despite these similarities, the prey association differed between years: in warm 2018, recognized sets of prey items were further characterized by a high contribution of *zoea* larvae of *Pagurus* spp., whereas this prey item remained relatively low in cold 2021. In contrast, neither mixed-regime 2024 nor warm 2023 showed prey associations that included *C. glacialis*. Instead, both years were characterized by low abundance of *C. finmarchicus* CIV/CV but differed in how diets were supplemented by an alternative prey. In 2024, low *C. finmarchicus* CIV/CV occurred either in combination with low *zoea* larvae of *Pagurus* spp. or with high contribution of Remaining prey, whereas in 2023 diets were mainly complemented by high abundance of *zoea* larvae of *Pagurus* spp. together with lower contributions of Remaining prey. The *furcilia* larvae of *Euphausiacea* was never detected in any recognized sets of prey items.

### Factors affecting at-sea *Calanus* spp. abundance in foraging areas of Little Auks

**Fig. 7:**
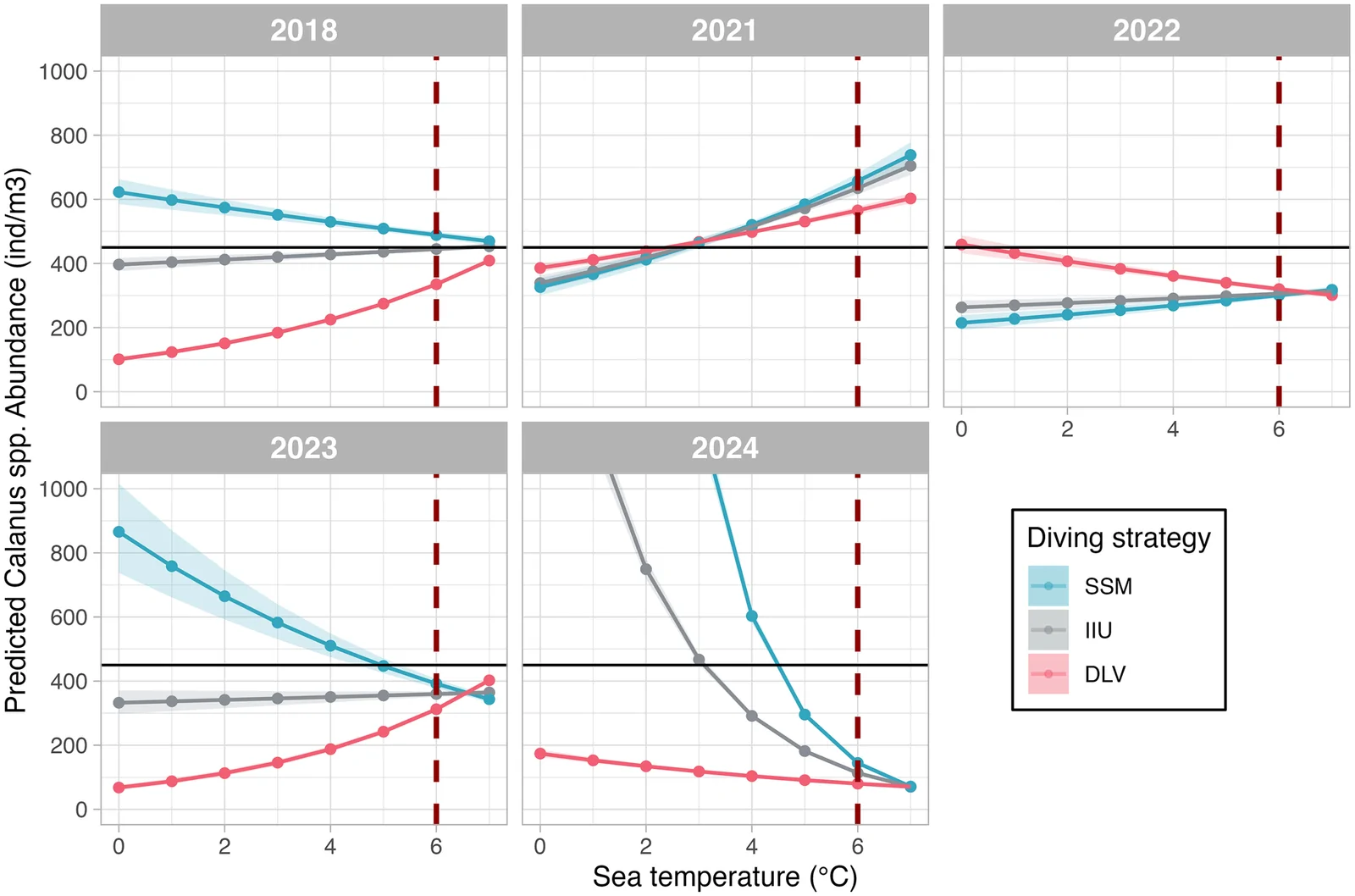
GLM-model based prediction of *Calanus* spp. abundance at three constant depths representing the mean foraging depth of the particular dive types (SSM, IIU and DLV) adopted by the TDR-tracked chick-rearing Little Auks for each studied years, based on LOPC-CTD data collected in their foraging areas. The vertical brown dashed lines represent a 6 °C physiological upper threshold for *Calanus glacialis* functioning in Svalbard [66]. The black lines indicate a 450 copepods · m^−3^ threshold representing possible minimal Little Auks foraging thresholds. The dots represent the temperature values used for prediction of *Calanus* spp. abundance at foraging depths (lines) representing the three main dive types in each studied year (SSM_2018_ = 6.5 m, SSM_2021_ = 3.9 m, SSM_2022_ = 5.0 m, SSM_2023_ = 4.7 m, SSM_2024_ = 4.7 m, IIU_2018_ = 11.4 m, IIU_2021_ = 9.8 m, IIU_2022_ = 13.5 m, IIU_2023_ = 13.1 m, IIU_2024_ = 11.9 m, DLV_2018_ = 26.2 m, DLV_2021_ = 29.7 m, DLV_2022_ = 37 m, DLV_2023_ = 27.2 m, DLV_2024_ = 22.2 m)

The highest ranked GLM (Supplementary Materials Table S5) revealed that the at-sea *Calanus* spp. abundance was significantly affected by year (GLM, χ^2^ = 10,582.88; *p* < 0.0001), depth (χ^2^ = 823.24; *p* < 0.0001), and the year × depth × temperature interaction (χ^2^ = 7,589.9; *p* < 0.0001) but not by the temperature alone (χ^2^ = 2.38; *p* = 0.123). Moreover, the zero-inflation component indicated that the probability of structural zeros (real absence of the prey because of non-optimal environmental conditions) decreased significantly with depth (estimate ± SE = −0.026 ± 0.0007, *p* < 0.001) and temperature (−0.155 ± 0.005, *p* < 0.001). The previous results suggest increasing probability of *Calanus* spp. presence with increasing water temperature and depth. *Post-hoc* comparison of the temperature slopes at specific depths among years revealed significant differences (all p **<**0.0001) with the exception of intermediate depths (ranges corresponding to IIU) between warm 2018 and mixed-regime 2022 (estimate = −0.004, *p* = 0.98), and deeper depths (ranges corresponding to DLV) between mixed-regime years 2022 and 2024 (estimate = −0.01, *p* = 0.29) (Supplementary Materials Table S7). The prediction of *Calanus* spp. abundance at shallow diving depths (SSM) in various temperatures based on GLM revealed that abundance of *Calanus* spp. decreased with increasing sea water temperature (warm 2018 and 2023, and mix-regime 2024). Moreover, in warm 2018 and 2023, *Calanus* spp. abundance at deeper depths (DLV range) increased with increasing temperature. In 2023 the highest *Calanus *spp. abundance was predicted at shallower depths (SSM) below 6 °C, corresponding to the physiological threshold of *C. glacialis*. In cold 2021, on the contrary, *Calanus* spp. abundance increased with increasing temperature regardless of the depth. In mixed-regime 2022 *Calanus* spp. abundance was relatively low with the highest abundance at the deepest depths (Fig. 7).

### Little Auks and *Calanus* spp. niche overlap

**Fig. 8:**
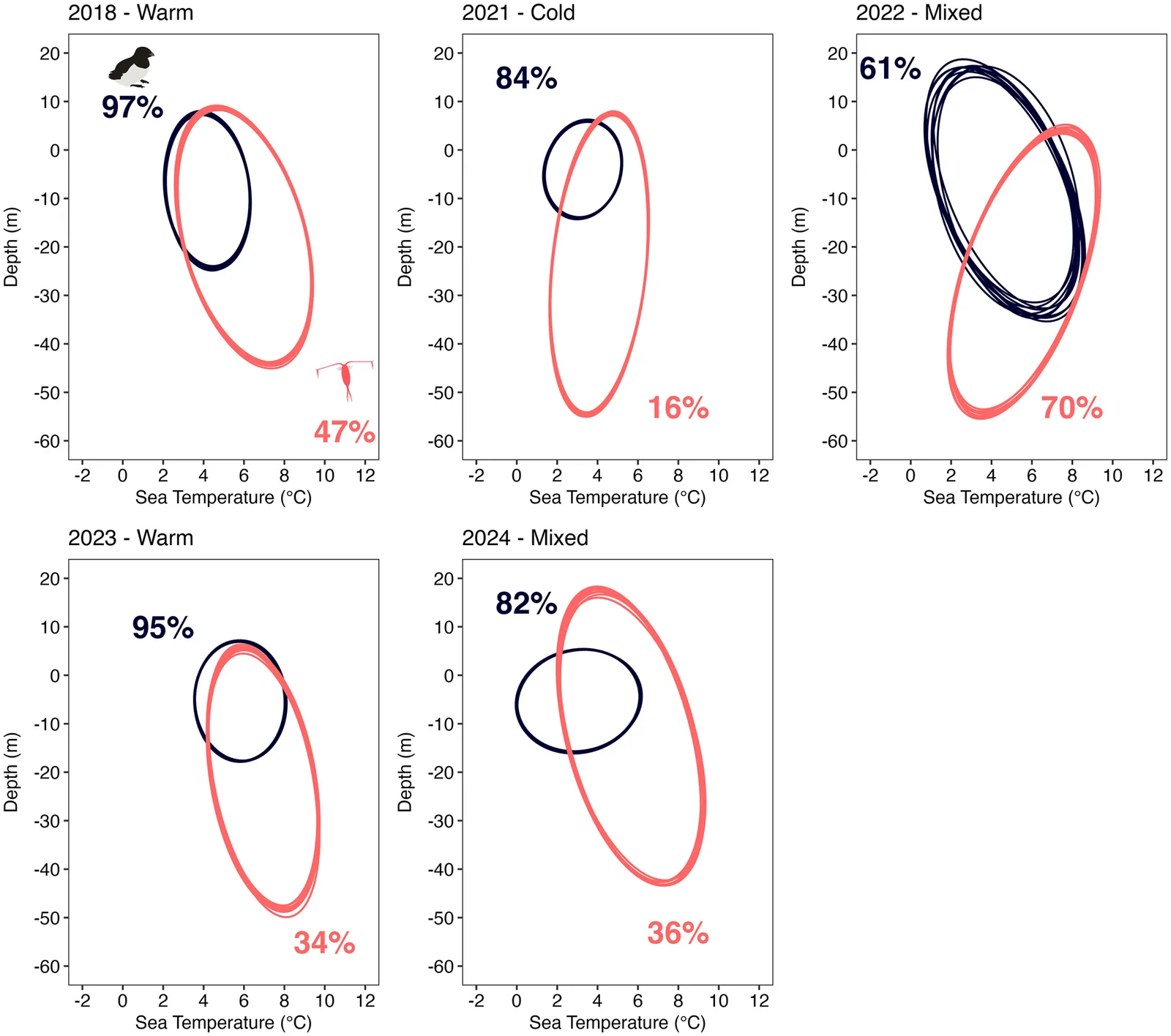
Ellipses of the environmental niches used by Little Auks and *Calanus* spp. over 5 years of study (2018–2024) representing ten randomly sampled projections of the niches in two‑dimensional perspectives of sea temperature [°C] and depth [m]. The individual overlap probability in environmental parameters use by the predator (Little Auk, black font and black ellipses) and the prey (*Calanus* spp., red font and red ellipses). The overall overlap when combining the niches of both the Little Auks and *Calanus* spp. is shown in the bottom right panel. Note: depths values > 0 m recorded for *Calanus* spp. in multiple years reflect the statistical spread of the data around the estimated mean

We recorded that the individual overlap (calculated in the *nicheROVER* package) of the Little Auk foraging niche with the environmental niche of *Calanus* spp. was high in most years, i.e. over 80% (97% in 2018, 84% in 2021, 95% in 2023, and 82% in 2024) except for mixed-regime 2022 (61%) (Fig. 8). We found that the maximum likelihood overlap (calculated in the *SIBER* package) of Little Auks and *Calanus* spp. niches (described by sea temperature and depth) varied between 16% (cold 2021) and 34% (warm 2018) (Fig. 8).

## Discussion

In this multidisciplinary study, combining GPS-TDR tracking of birds with at-sea zooplankton surveys using LOPC-CTD, and diet analyses, we showed that chick-rearing Little Auks nesting in one of the largest breeding aggregations of this species in Svalbard maintained a consistent foraging strategy in response to fluctuating environmental conditions over three of five years of the study period. They slightly adapted their dive types and prey capture in the other two years.

We found that the diving depth varied with respect to environmental conditions with Little Auks employing three distinct dive types, particularly in two years (2018 and 2022) (Fig. 6). SSM dives (Shallow dives, Short duration, Mix dive shape) were the most common, representing 94.8% of all the dives recorded over all the study years. DLV dives (3.7%) and IIU dives (1.5%) were much less frequent. However, when looking at the yearly proportion of DLV dives, they respectively represented 8.3% and 9.6% of the dive types in 2018 and 2022. A comparable pattern, with performing short and shallow dives as well as deep, long dives has also been recorded in the Cassin’s Auklet (*Ptychoramphus aleuticus*), a Pacific zooplanktivorous alcid similar in size to the Little Auk [97]. However, Cassin’s Auklets have performed short, shallow dives in years of low main prey (krill) density and deep dives in years of high main prey density, while Little Auks exhibited a more complex pattern. In our study, short, shallow dives occurred in higher proportions during cold 2021, warm 2023 and mixed-regime 2024, while deeper, longer dives occurred during warm 2018 and mixed-regime 2022 (Fig. 6). Differences in the diving type of both alcid species likely stem from their geographic location. Cassin’s auklets breed at lower latitudes, attend their colony at night to avoid predation, and feed primarily in the upwelling zone of the California Current Systems. In contrast, Little Auks occur in the High Arctic and forage in summer during continuous polar daylight. Distinct dive types have also been recognized in Little Auks breeding in East Greenland, with shallow and deep categories distinguished. Deep dives were probably dedicated for preying on deep water *C. hyperboreus* representing 47–65% of prey abundance. Shallower dives predominated in conditions of higher ice concentration and were probably focused on ice-associated zooplankton [98]. Those conditions are not fully comparable with the Hornsund area, with no sea ice available in cost-effective distance from the colony during our study years, and relatively low proportion of *C. hyperboreus* in the diet (0–0.2% during this study) [37]. Since IIU dives only represented a fraction of dives, we hypothesize that they represent diverted DLV dives when birds opportunistically encountered an attractive prey patch while descending to the deeper water column layers and subsequently foraged on it.

We found that despite inter-annual variability of environmental conditions the three dive types remained present over the years studied. We recommend using the SOM model to classify diving metrics. It can benefit the detection of more sophisticated dive patterns dedicated to exploring particular foraging microhabitats in other seabird species. This technique is based on unsupervised machine learning. Therefore, it is not biased by arbitrary cut-off points proposed by the observer. Nevertheless, the range of main diving metrics, such as the mean foraging depth or mean dive duration, fluctuated from year to year. This variation in mean foraging depths among years indicates that the vertical availability of prey patches fluctuates [17, 97]. Little Auks may have followed the shifts in the distribution of their preferred prey, which are driven by spatial and temporal variation in environmental conditions. This could explain the observed interannual differences in dive depth within the same dive type. However, we were unable to link those changes in diving depth to particular *Calanus* species due to the large size overlap of both prey species [36, 51, 86, 87]. At the finer temporal scale (within a season), however, dives occurring within a similar radius were more similar, suggesting that birds repeatedly targeted the same prey patches and therefore used similar dive types. Such spatio-temporal consistency in dive behaviour has also been reported in other seabirds, including Brünnich’s Guillemots and Little Penguins *Eudyptula minor *[19, 99]). In Brünnich’s Guillemots, distinct dive types have been linked to the capture of specific prey items, further supporting the idea that local environmental conditions and prey distribution shape foraging strategy [19].

Over the years, the sea water properties in the foraging areas fluctuated, forcing Little Auks to adopt different foraging strategies depending on water properties. Behavioral flexibility is crucial for thriving in the era of rapid climate change. The long-term climate change-related changes in oceanographic environment and marine food web led to extirpation of southernmost population Little Auks in Iceland [100] and some colonies on the western coast of Greenland [101].

We expected that Little Auks would adopt an **optimal-foraging strategy** in prevalence of cold-water masses, an **effort-foraging strategy** when warm water masses predominated, and a **reactive-foraging strategy** when water conditions are represented by the mixed regime. We found that optimal-foraging strategy was used by Little Auks during the most optimal year (cold 2021), the least optimal year (warm 2023) but also during the mixed-regime 2024. It demonstrates that despite contrasting foraging conditions, Little Auks were able to remain consistent in the foraging strategy they adopted. In cold 2021, a broad layer of cold-water patches was available to Little Auks (Fig. 2, Supplementary Material Fig S6), allowing them to forage on their preferred prey, *C. glacialis* and performing mostly SSM dives (Fig. 6). Despite these favorable conditions, this year was characterized by the lowest overall overlap between Little Auks and *Calanus* spp. niches (Fig. 8). This apparent contradiction is consistent with findings from other seabird studies (e.g., guillemots, Rhinoceros Auklets) [102–104]. These studies demonstrated that predator-prey spatial overlap tended to increase when prey-density was intermediate, as predators aggregated their foraging effort in few available high-density patches. Conversely, when prey is abundant, predators may not need to target areas of peak density, as foraging conditions are sufficiently favorable across a broader area. It is also important to stress that Little Auks behave as a central-place forager during the breeding season. Thus, the duration and distance of their foraging trips are bound by their need to return to the colony to feed their chick, thus birds could not always travel to the best locations for prey [21]. Moreover, their choice of foraging areas is constrained by conditions that affects prey accessibility; for instance, strong winds can prevent little auks, which rely on costly flapping flights, from reaching good feeding patches [52]. In mixed-regime 2024 the optimal-foraging strategy was adopted due to the high availability of *Calanus* spp. in the shallow cold waters (Fig. 6) located in the close vicinity of the colony which remained available throughout the season (Fig. 2).

In the warmest 2023, cold-water patches were scarce in the surface layer (Fig. 2), yet birds still prioritized foraging there and did not shift to a costly **effort-foraging strategy** by diving deeper. On one hand, increasing diving depth for all the dives would require more energy, as the birds must overcome water drag and buoyancy to reach greater depths [8, 14] or is limited by the oxygen store of the bird [105]. On the other hand, increasing diving depth may not be necessary if local ecological variation (i.e. prey availability in shallow water) allows the birds to keep foraging at their typical depth [106]. Their ability to maintain SSM dives was possible as they supplemented their diet with alternative prey, such as *furcilia* larvae of *Euphausiacea* [37]. It illustrates a refined form of optimal-foraging strategy that minimizes energy expenditure under poor conditions. Due to increasing water temperature, the population of *Euphausiacea* has been increasing in the waters surrounding Svalbard over the last two decades [107]. Few studies have investigated the energy content of *Euphausiacea* in the Arctic [108] and to our knowledge, none have investigated the energy content of the *furcilia* larval stage of *Euphausiacea*. However, given that the energy content of the adult euphausiids is quite high – [26.75–22.71 kJ · g^−1^ dry weight [108]], we suggest that the *furcilia* larvae might represent a valuable alternative prey for Little Auks. The CBA identified mostly warm water prey associations in food samples collected in warm 2023 (Table 2), indicating a lack of sufficient optimal foraging patches, as well as high prey heterogeneity in the available food patches that year. This may have led individuals to capture whatever they could find in the exploited foraging patch. Diet switching or supplementing with complementary prey are not uncommon in seabirds, particularly under rapid ocean warming [109, 110]. For instance, Rhinoceros Auklets (*Cerorhinca monocerata*) have been observed shifting from cold-water to warm-water prey species in response to changing marine conditions [109]. Similarly, the study on Cassin’s Auklets has revealed that they adjust the proportion of copepods, euphausiids, and larval fish they deliver to their chicks based on oceanographic conditions. However, the mentioned study also has revealed that different prey items have different energetic values: copepods support faster chick growth, whereas larval fish result in slower growth [5]. Thus, switching or supplementing diets can serve as an adaptive response to environmental change and prey community borealization, but with increased presence of temperate prey in the marine Arctic food web, chicks are expected to fledge at lighter weights, reducing their chances of survival.

We found that studied birds adopted a **reactive-foraging strategy** in warm 2018 and mixed-regime 2022, both years characterized by patchy distribution of various SST masses (Fig. 2). In both years, birds concentrated most dives in cold waters and performed SSM dives (Figs. 4, 6). However, as the proportion of suboptimal (transitional or warm) waters increased, so did the proportion of DLV dives (Fig. 6). This diving behavior flexibility also extended to the prey choice: alongside their preferred *C. glacialis*, Little Auks incorporated to the chick diet warm-water *C. finmarchicus* and *zoea* larval stage of hermit crab *Pagurus* spp. (Table 2). In contrast to *C. finmarchicus* occurring in the Hornsund area and the surrounding waters of southern Spitsbergen in the upper layer of the water column [17], the highest concentrations of adults and larvae hermit crabs (*Pagurus* spp.) have been found at depths between 20 and 30 m [55, 110], i.e. the depth matching with the range of Little Auks DLV dives in all years. With increasing water temperature both mentioned prey species are expanding their repartition into Arctic waters [55, 110], and have been detected in the Little Auks diet, albeit irregularly, since at least 2011 [37].

Mixed-regime 2022 was particularly different from the other years studied, with the lowest niche overlap between Little Auks and *Calanus* spp. (61%) (Fig. 8). Mixed-regime 2022 was also characterized by the lowest abundance of *Calanus* spp. in shallow waters, regardless of water temperature. The highest *Calanus* spp. abundances were recorded at deeper depths; however, even at these depths, densities remained low, averaging only 300 copepods · m^−3^ (Fig. 7).

Although sea temperature can help explain the vertical distribution of *Calanus* spp. in the water column, it did not appear to account for the low *Calanus* spp. abundance observed in 2022, suggesting that other factors were involved. In this context, it is important to consider that during the summer polar day zooplankton do not perform diel vertical migration [15], yet individuals can still adjust their position in response to local conditions. Zooplankton may respond to multiple drivers, including light conditions, predation risk, and physical forcing such as meltwater-driven turbidity, which can in turn influence their distribution and food availability [111]. One possible explanation therefore lies in bottom-up processes, as *Calanus* spp. are primarily herbivorous on the shelf [35]. In 2022, chlorophyll-a concentration was low in the Hornsund area [37], potentially limiting food resources for *Calanus* spp.; however similar chlorophyll-a concentrations were also recorded in 2021, which was consider an optimal year for Little Auks [37], indicating that this factor alone is unlikely to explain low *Calanus* spp. density.

Longer-term processes should also be considered, particularly the influence of sea-ice conditions on early life stages of *C. glacialis*, which require up to two years to reach the CV stage. For instance, [112] have reported a positive relationship between breeding success of Little Auks and sea ice concentration with a two-year lag. Similarly, [100] and [53] have recorded time-lags effect of environmental variables on the population size and diets of Little Auks. In this context, disentangling the drivers of the low *Calanus* spp. abundance observed in 2022 remains challenging.

Little Auks performed the deepest DLV dives of all studied years in 2022, reaching an average depth of 37.1 m. This suggests that birds were targeting deeper water layers with higher abundance of prey. Such behavior may reflect a response to limited prey availability in shallower waters. When prey density falls below a certain threshold, birds may be unable to capture enough prey to meet their energetic requirements. In turn, they are forced to dive deeper to maintain their energy intake, but at the cost of higher energy expenditure. Prey abundance thresholds during foraging have been observed in Double-crested Cormorants *Phalacrocorax auritus* [113] showing disproportionately reduced capture efficiency below 2 g of prey · m^−3^. In contrast, lower abundance of prey was associated with an increase capturing rate in Peruvian Booby *Sula variegata* and Guanay Cormorant *Phalacrocorax bougainvilliorum *[114]. Thus, while Little Auks demonstrated notable flexibility in dive behavior and prey use, their reliance on cold, copepod-rich habitats sets clear ecological limits. When warming reduces densities of the preferred prey in shallow waters under a certain threshold, the costs of deeper, effort-intensive foraging may outweigh the birds’ capacity to compensate.

Our study had several limitations that should be considered when interpreting the results. First, we based our assessment of Little Auk dives on GPS-TDR loggers. Birds may not have behaved naturally, with a potential increase in their spatial foraging range compared to non-tagged individuals [115, 116]. However, since the same type of loggers were used across all seasons, any potential bias was likely consistent throughout the years. To reduce their stress burden, food samples were not collected from tagged individuals. Second, due to logistic difficulties we were not able to organize the at-sea LOPC-CTD measurements from the research vessel at the same time as the GPS-TDR-tracking of Little Auks. Given the high dynamics of the marine ecosystem, *Calanus* spp. abundance and vertical distribution may differ from the state encountered by the GPS-TDR tracked Little Auks while they were foraging. On the other hand, the surveyed area has specific oceanographic features, where water masses follow the bottom topography, stabilizing the Polar Front along the shelf break and structuring both the distribution of water masses [92] and the associated cold- and warm- water zooplankton communities [53]. Thus, we think that the LOPC-CTD data were likely representative of the general vertical pattern of *Calanus* spp. distribution during the Little Auks’ chick-rearing period. Despite these limitations, our study provides in-depth insight into Little Auk foraging ecology, showing real foraging depth and associated temperatures.

## Conclusions

Of the three anticipated foraging strategies, Little Auks employed only the **optimal-foraging** and the **reactive-foraging strategy**. This demonstrates the birds’ ability to switch between strategies depending on prey availability and conditions in water masses. The **optimal-foraging strategy** was employed during environmentally contrasted years, e.g., in cold 2021, warm 2023, and mixed-regime 2024, while birds in warm 2018 and mixed-regime 2022 adopted a **reactive-foraging strategy**. As the environmental conditions in 2021 and 2023 were quite different (cold vs warm), in warm 2023 Little Auks adopted a refined version of the optimal-foraging strategy by supplementing the diet with alternative prey. This strategy of supplementing chick diet with alternative prey can be considered as an adaptive response to changes in the prey community due to an increase in sea temperatures. However, supplementary prey may not always be as energy rich as the preferred *C. glacialis*, which could affect the population’s long-term viability. Continued reliance on lower-energy prey raises concerns about whether such flexibility can support the population under ongoing climate change. Continued long-term monitoring is therefore essential to determine whether this behavioral flexibility will remain sufficient under future climate conditions.

## Electronic supplementary material

Below is the link to the electronic supplementary material.

Supplementary material 1

## Data Availability

The data necessary for replicating the analyses are available in OSF repository: https://osf.io/7erbc/overview?view_only=3fbdda3c1b9b4bfea47685b0d13263e9.

## Abbreviations

TDR-GPS: Temperature depth recorder – global positioning system
LOPC-CTD: Laser optical plankton counter – conductivity temperature depth
SOM: Self-organizing map
CIT: Conditional inference tree
INLA: Integrated nested Laplace approximation
CBA: Classification based on association model
FTC: Foraging thermal categories
SST: Sea surface temperature
DIC: Deviance information criterion
AIC: Akaike information criterion
CI: Credible intervals
GLM: Generalized linear model
SSM: Shallow dives with short duration and mix of U- and V-shape
IIU: Intermediate dives with intermediate duration and U-shape
DLV: Deep dives with long duration and V-shape
*C. glacialis* CV: Copepodite developmental stage of *Calanus glacialis*
