# Supplementary material for: Diving ecology of a pelagic seabird, the Little Auk Alle alle, in a changing Arctic revealed by multiyear tracking and optical zooplankton data
Source: Mov Ecol. 2026 Jul 23;14:73. doi: 10.1186/s40462-026-00679-1 (PMC13681773; doi:10.1186/s40462-026-00679-1)
Supplement: Supplementary file 1 [file 40462_2026_679_MOESM1_ESM.docx]

**Supplementary Materials**

**Diving Ecology of a Pelagic Seabird, the Little Auk *Alle alle,* in a Changing Arctic Revealed by Multi‑Year Tracking and Optical Zooplankton Data**

Pauline Bodson^1, *^, Kaja Balazy^2^, Katarzyna Błachowiak-Samołyk^2^, Rafał Boehnke^2^, Lech Marek Iliszko^1^, Kristin Piening^1^, Martyna Syposz^1^, Lech Stempniewicz^1^, Emilia Trudnowska^2^, Katarzyna Wojczulanis-Jakubas^1^, Dariusz Jakubas^1, *^

**Figure S1.** An example of dives recorded for the Little Auk equipped with a GPS-TDR logger (logger ID: ADD09) in 2018 with information about the foraging temperature (upper panel) and the depth (lower panel) recorded by the TDR logger. Phases of dives: D = descent, DB = descent/bottom, B = bottom, BA = bottom/ascent and A = ascent. Dives 1, 4, 5, 6 represent U-shaped dives, whereas dives 2 and 3 represent V-shaped dives (see description in the main text). The red circle matches the area that is the deepest phase of the dive representing the foraging phase. The graph was generated in the *diveMove* package (Luque, 2024).

**Figure S2.** Home range (95% autocorrelated kernel density) of foraging position of GPS-TDR tracked Little Auks breeding in Hornsund, SW Spitsbergen (colored area), and transects of laser optical plankton counter (LOPC) combined with Conductivity, Temperature, and Depth (CTD) unit. Background: bathymetric contours. Home ranges (95% autocorrelated kernel density) calculated using the *akde* function in the *ctmm* package (Fleming & Calabrese, 2023). Basic and bathymetry maps were generated in the *ggOceanMaps* package (Vihtakari, 2024)

**Figure S3.** Map of INLA SPDE (Integrated Nested Laplace Approximation Stochastic Partial Differential Equation) predicted spatial distribution of foraging depth of Little Auks breeding in Hornsund in 2021(A) and 2023 (B) (two years with sufficient sample size for analyses). The green diamond represents the location of the Little Auk colony. The gradient scale represents the diving depth [m] predicted by INLA SPDE. The blue contours mark the 100 m (light blue), 150 m (medium blue), and 200 m (dark blue) isobath. The hollow white circles represent the GPS locations of dives.

**Figure S4.** Map of INLA SPDE (Integrated Nested Laplace Approximation Stochastic Partial Differential Equation) predicted spatial distribution of foraging temperature [ºC] of Little Auks breeding in Hornsund in 2021(A) and 2023 (B) (two years with sufficient sample size for analyses). The green diamond represents the location of the Little Auk colony. The gradient scale represents the predicted foraging temperature. The blue contours mark the 100 m (light blue), 150 m (medium blue), and 200 m (dark blue) isobath. The hollow white circles represent the GPS locations of dives.

**Figure S5.** Annual environmental niches represented by sea temperature [°C] and depth [m] utilized by Little Auks and *Calanus* spp*.* over 5 years of study (2018-2024). Distribution of foraging depth (A) and temperature (B) of Little Auks, and ellipses representing ten random projections of the niches in two‑dimensional perspectives of two variables. Distribution of depth (D), sea temperature (E) and *Calanus* spp. abundance (F) recorded by the LOPC-CTD in the Little Auks foraging areas, and ellipses representing ten random projections of the niches in two‑dimensional perspectives of two variables [*Calanus* spp. abundance and sea temperature (G) and *Calanus* spp. abundance and depth (H)]. Graphs were created in the *nicheROVER* package (Lysy et al., 2023). Little Auk and zooplankton logo by Quentin Gallot.

**Figure S6.** The LOPC-CTD profile (see location in Supplementary Material Fig. S2) showing the water temperature and *Calanus* spp. abundance over five years in the Hornsund area. The isolines represent water temperature at different depths within the water column. The background color indicates the total abundance of *Calanus* spp. The shaded area below 40 m represents depths not reachable by diving Little Auks. The remaining shaded area represents water masses with a temperature higher than 6°C, the tolerance threshold for the Little Auks' preferred prey, the cold-water copepod, *Calanus glacialis*. The non-shaded area represents the area where Little Auks and *Calanus glacialis* can overlap.

| **Dive shape** | DLV | | SSM | | IIU | |
| --- | --- | --- | --- | --- | --- | --- |
| Comparison | F Model | P Value | F Model | P Value | F Model | P Value |
| 2018 vs 2021 | 0.20 | > 0.05 | 26.75 | **<0.001** | 14.82 | **<0.001** |
| 2018 vs 2022 | 1.46 | > 0.05 | 76.90 | **<0.001** | 0.99 | 0.3226 |
| 2018 vs 2023 | 0.29 | > 0.05 | 278.82 | **<0.001** | 2.88 | 0.0908 |
| 2018 vs 2024 | 0.22 | > 0.05 | 217.39 | **<0.001** | 15.58 | **<0.001** |
| 2021 vs 2022 | 6.89 | > 0.05 | 52.66 | **<0.001** | 0.84 | 0.3832 |
| 2021 vs 2023 | 0.19 | > 0.05 | 120.05 | **<0.001** | 17.19 | **<0.001** |
| 2021 vs 2024 | 1.84 | > 0.05 | 86.92 | **<0.001** | 0.37 | 0.5452 |
| 2022 vs 2023 | 3.54 | > 0.05 | 24.34 | **<0.001** | 1.99 | 0.166 |
| 2022 vs 2024 | 9.27 | > 0.05 | 26.39 | **<0.001** | 0.11 | 0.7478 |
| 2023 vs 2024 | 2.28 | > 0.05 | 1.23 | 0.2736 | 13.59 | **<0.001** |

**Table S1.** Results of the permutational analysis of variance (PERMANOVA) for each diving parameter of the GPS-TDR-tracked chick rearing Little Auks breeding in Hornsund, SW Spitsbergen (bottom frequency, dive duration [sec], foraging depth [m]) and dive temperature on each dive type (DLV, IIU, and SSM) comparing the 5 years of study. Bolded p values are significant.

| **Dive duration**  **[sec]** | DLV | | SSM | | IIU | |
| --- | --- | --- | --- | --- | --- | --- |
| Comparison | F Model | P Value | F Model | P Value | F Model | P Value |
| 2018 vs 2021 | 1.46 | 0.2396 | 2,770.28 | **<0.001** | 1.46 | 0.2278 |
| 2018 vs 2022 | 65.96 | **<0.001** | 85.07 | **<0.001** | 28.01 | **<0.001** |
| 2018 vs 2023 | 7.38 | 0.0056 | 551.84 | **<0.001** | 1.48 | 0.2314 |
| 2018 vs 2024 | 73.69 | **<0.001** | 1,472.03 | **<0.001** | 52.73 | **<0.001** |
| 2021 vs 2022 | 38.01 | **<0.001** | 79.63 | **<0.001** | 30.03 | **<0.001** |
| 2021 vs 2023 | 0.00 | 1 | 1,146.07 | **<0.001** | 0.14 | 0.7218 |
| 2021 vs 2024 | 23.43 | **<0.001** | 649.93 | **<0.001** | 3.43 | 0.0708 |
| 2022 vs 2023 | 46.67 | **<0.001** | 1.07 | 0.2958 | 15.81 | **<0.001** |
| 2022 vs 2024 | 118.33 | **<0.001** | 7.61 | **0.0068** | 14.94 | **<0.001** |
| 2023 vs 2024 | 93.46 | **<0.001** | 144.97 | **<0.001** | 16.32 | **4e-04** |

| **Foraging depth [m]** | DLV | | SSM | | IIU | |
| --- | --- | --- | --- | --- | --- | --- |
| Comparison | F Model | P Value | F Model | P Value | F Model | P Value |
| 2018 vs 2021 | 12.75 | **<0.001** | 1,035.64 | **<0.001** | 3.17 | 0.0774 |
| 2018 vs 2022 | 123.89 | **<0.001** | 40.34 | **<0.001** | 2.15 | 0.1484 |
| 2018 vs 2023 | 5.79 | **0.0134** | 680.92 | **<0.001** | 14.05 | **<0.001** |
| 2018 vs 2024 | 73.95 | **<0.001** | 651.38 | **<0.001** | 0.88 | 0.3656 |
| 2021 vs 2022 | 38.95 | **<0.001** | 19.85 | **<0.001** | 3.98 | 0.0708 |
| 2021 vs 2023 | 8.55 | **0.0038** | 131.22 | **<0.001** | 8.87 | **0.0046** |
| 2021 vs 2024 | 57.18 | **<0.001** | 117.08 | **<0.001** | 2.43 | 0.1174 |
| 2022 vs 2023 | 124.11 | **<0.001** | 1.98 | 0.1576 | 0.06 | 0.833 |
| 2022 vs 2024 | 209.48 | **<0.001** | 2.29 | 0.1378 | 0.59 | 0.4462 |
| 2023 vs 2024 | 105.96 | **<0.001** | 0.06 | 0.7986 | 2.69 | 0.0978 |

| **Foraging temperature [°C]** | DLV | | SSM | | IIU | |
| --- | --- | --- | --- | --- | --- | --- |
| Comparison | F Model | P Value | F Model | P Value | F Model | P Value |
| 2018 vs 2021 | 54.55 | **<0.001** | 2,241.00 | **<0.001** | 5.21 | **0.0248** |
| 2018 vs 2022 | 44.94 | **<0.001** | 21.88 | **<0.001** | 52.75 | **<0.001** |
| 2018 vs 2023 | 149.70 | **<0.001** | 7687.12 | **<0.001** | 196.70 | **<0.001** |
| 2018 vs 2024 | 232.40 | **<0.001** | 2,084.77 | **<0.001** | 0.87 | 0.3428 |
| 2021 vs 2022 | 114.59 | **<0.001** | 405.78 | **<0.001** | 37.30 | **0.0018** |
| 2021 vs 2023 | 174.18 | **<0.001** | 19,523.15 | **<0.001** | 68.65 | **<0.001** |
| 2021 vs 2024 | 0.15 | 0.7026 | 93.73 | **<0.001** | 0.80 | 0.385 |
| 2022 vs 2023 | 2.00 | 0.154 | 459.65 | **<0.001** | 7.28 | **0.0104** |
| 2022 vs 2024 | 127.40 | **<0.001** | 258.34 | **<0.001** | 16.77 | **<0.001** |
| 2023 vs 2024 | 499.05 | **<0.001** | 16,651.09 | **<0.001** | 67.17 | **<0.001** |

**Table S2.** Summary (mean ± SD) of the main Little Auks dive metrics: dive shape, dive duration [sec], foraging depth [m], and foraging temperature [°C] for each dive type: SSM (Shallow dives, Short duration, Mix dive shape), IIU (Intermediate depth, Intermediate duration, U-shaped dives) and DLV (Deep dives, Long duration, V-shaped dives) among the 5 years of study. Inter-annual significant differences within a given dive type depicted with different subscripted letters (PERMANOVA, p < 0.05).

| Diving type & Year | No of dives | Dive duration [sec] | Foraging depth [m] | Dive shape | Foraging temperature [°C] |
| --- | --- | --- | --- | --- | --- |
| SSM - 2018 | 3,488 | 37.4 ± 10.8^a^ | 6.5 ± 3.4^a^ | 0.300 ± 0.18^a^ | 4.15 ± 0.8^a^ |
| SSM - 2021 | 3,849 | 22.2 ± 13.6^b^ | 3.8 ± 3.6^b^ | 0.278 ± 0.18^b^ | 3.27 ± 0.79^b^ |
| SSM - 2022 | 223 | 30.5 ± 11.4^c^ | 5.0 ± 4.3^c^ | 0.190 ± 0.15^c^ | 4.43 ± 1.4^c^ |
| SSM - 2023 | 5,704 | 31.4 ± 12.6^c^ | 4.7 ± 3.2^c^ | 0.241 ± 0.15^d^ | 5.81 ± 0.92^d^ |
| SSM - 2024 | 4,557 | 28.7 ± 9.6^d^ | 4.7 ± 3.1^c^ | 0.244 ± 0.15^d^ | 3.05 ± 1.24^e^ |
| IIU - 2018 | 163 | 53.7 ± 6.5^a^ | 11.4 ± 2.9^a^ | 0.356 ± 0.09^a^ | 3.9 ± 0.78^a^ |
| IIU - 2021 | **11**† | 51.3 ± 7.0^ac^ | 9.8 ± 3.4^a^ | 0.469 ± 0.13^bc^ | 3.34 ± 0.72^b^ |
| IIU - 2022 | **4**† | 71.0 ± 1.2^b^ | 13.5 ± 1.6^ab^ | 0.403 ± 0.12_abc_ | 6.84 ± 1.56^c^ |
| IIU - 2023 | 59 | 52.4 ± 9.3^a^ | 13.1 ± 3.4^b^ | 0.332 ± 0.1^a^ | 5.6 ± 0.84^c^ |
| IIU - 2024 | 45 | 43.0 ± 14.4^c^ | 11.9 ± 4.2^ab^ | 0.434 ± 0.18^c^ | 3.75 ± 1.44^a^ |
| DLV - 2018 | 334 | 59.9 ± 6.9^a^ | 26.2 ± 4.6^a^ | 0.043 ± 0.06^a^ | 4.9 ± 1.06^a^ |
| DLV - 2021 | 23 | 61.7 ± 4.5^a^ | 29.7 ± 2.8^b^ | 0.038 ± 0.01^a^ | 3.25 ± 0.55^b^ |
| DLV - 2022 | 24 | 71.7 ± 6.4^b^ | 37.1 ± 5.0^c^ | 0.029 ± 0.01^a^ | 6.44 ± 1.32^c^ |
| DLV - 2023 | 171 | 61.6 ± 6.8^a^ | 27.2 ± 3.9^d^ | 0.041 ± 0.03^a^ | 6.11 ± 1.02^c^ |
| DLV - 2024 | 139 | 53.7 ± 7.6^c^ | 22.3 ± 4.6^e^ | 0.046 ± 0.03^a^ | 3.14 ± 1.32^b^ |

† Estimates based on fewer than 20 dives should be interpreted with caution.

**Table S3.** Pairwise effect sizes of Short, Shallow dives (SSM) compared between years for foraging depth, dive duration, dive shape, and foraging temperature using the Cohen’s d indices. Effect Size are classified as followed: Small: 0.2, Medium: 0.5, Large: 0.8 and Very Large: 1.3 (Sullivan & Feinn, 2012). CL – Confidence Limit. Effect size calculated using the *emmeans* package (Lenth, 2023).

| Diving variable | *Pairwise comparison* | *Effect size* | *SE* | *df* | *Lower CL* | *Upper CL* |
| --- | --- | --- | --- | --- | --- | --- |
| Foraging depth | SSM2018 - SSM2021 | 0.79 | 0.02 | 17859 | 0.75 | 0.84 |
|  | SSM2018 - SSM2022 | 0.46 | 0.07 | 17859 | 0.32 | 0.59 |
|  | SSM2018 - SSM2023 | 0.53 | 0.02 | 17859 | 0.49 | 0.58 |
|  | SSM2018 - SSM2024 | 0.56 | 0.02 | 17859 | 0.51 | 0.60 |
|  | SSM2021 - SSM2022 | -0.33 | 0.07 | 17859 | -0.47 | -0.20 |
|  | SSM2021 - SSM2023 | -0.26 | 0.02 | 17859 | -0.30 | -0.22 |
|  | SSM2021 - SSM2024 | -0.24 | 0.02 | 17859 | -0.28 | -0.19 |
|  | SSM2022 - SSM2023 | 0.08 | 0.07 | 17859 | -0.06 | 0.21 |
|  | SSM2022 - SSM2024 | 0.10 | 0.07 | 17859 | -0.04 | 0.23 |
|  | SSM2023 - SSM2024 | 0.02 | 0.02 | 17859 | -0.02 | 0.06 |
| Dive duration | SSM2018 - SSM2021 | 1.29 | 0.02 | 17859 | 1.24 | 1.34 |
|  | SSM2018 - SSM2022 | 0.59 | 0.07 | 17859 | 0.45 | 0.72 |
|  | SSM2018 - SSM2023 | 0.50 | 0.02 | 17859 | 0.46 | 0.54 |
|  | SSM2018 - SSM2024 | 0.74 | 0.02 | 17859 | 0.70 | 0.79 |
|  | SSM2021 - SSM2022 | -0.70 | 0.07 | 17859 | -0.84 | -0.57 |
|  | SSM2021 - SSM2023 | -0.79 | 0.02 | 17859 | -0.83 | -0.75 |
|  | SSM2021 - SSM2024 | -0.55 | 0.02 | 17859 | -0.59 | -0.50 |
|  | SSM2022 - SSM2023 | -0.09 | 0.07 | 17859 | -0.22 | 0.05 |
|  | SSM2022 - SSM2024 | 0.15 | 0.07 | 17859 | 0.02 | 0.29 |
|  | SSM2023 - SSM2024 | 0.24 | 0.02 | 17859 | 0.20 | 0.28 |
| Dive shape | SSM2018 - SSM2021 | 0.13 | 0.02 | 17859 | 0.09 | 0.18 |
|  | SSM2018 - SSM2022 | 0.67 | 0.07 | 17859 | 0.53 | 0.80 |
|  | SSM2018 - SSM2023 | 0.36 | 0.02 | 17859 | 0.31 | 0.40 |
|  | SSM2018 - SSM2024 | 0.34 | 0.02 | 17859 | 0.29 | 0.38 |
|  | SSM2021 - SSM2022 | 0.54 | 0.07 | 17859 | 0.40 | 0.67 |
|  | SSM2021 - SSM2023 | 0.22 | 0.02 | 17859 | 0.18 | 0.26 |
|  | SSM2021 - SSM2024 | 0.21 | 0.02 | 17859 | 0.16 | 0.25 |
|  | SSM2022 - SSM2023 | -0.31 | 0.07 | 17859 | -0.45 | -0.18 |
|  | SSM2022 - SSM2024 | -0.33 | 0.07 | 17859 | -0.46 | -0.20 |
|  | SSM2023 - SSM2024 | -0.02 | 0.02 | 17859 | -0.06 | 0.02 |
| Foraging temperature | SSM2018 - SSM2021 | 0.95 | 0.02 | 15629 | 0.90 | 1.00 |
|  | SSM2018 - SSM2022 | -0.30 | 0.07 | 15629 | -0.43 | -0.16 |
|  | SSM2018 - SSM2023 | -1.73 | 0.02 | 15629 | -1.77 | -1.68 |
|  | SSM2018 - SSM2024 | 0.82 | 0.03 | 15629 | 0.77 | 0.87 |
|  | SSM2021 - SSM2022 | -1.25 | 0.07 | 15629 | -1.38 | -1.11 |
|  | SSM2021 - SSM2023 | -2.68 | 0.03 | 15629 | -2.73 | -2.63 |
|  | SSM2021 - SSM2024 | -0.13 | 0.03 | 15629 | -0.18 | -0.08 |
|  | SSM2022 - SSM2023 | -1.43 | 0.07 | 15629 | -1.57 | -1.30 |
|  | SSM2022 - SSM2024 | 1.12 | 0.07 | 15629 | 0.98 | 1.26 |
|  | SSM2023 - SSM2024 | 2.55 | 0.03 | 15629 | 2.49 | 2.60 |

**Table S4.** Comparison of Integrated Nested Laplace Approximation Stochastic Partial Differential Equation (INLA) fitted models based on the Deviance Information Criterion (DIC). The lowest DIC corresponds to the best model (bolded). When the difference in DIC is lower than 7, we consider the model equivalent and chose the simplest model. rw 2 – smoothing parameter (second-order random walk latent effect), iid – random effect (independent and identically distributed random effects), spde – spatial field

| **Year** | **Model number** | **Response variable** | **Explanatory Variables** | **DIC** |
| --- | --- | --- | --- | --- |
| 2021 | mD_1_2021_ | Foraging depth | Bathymetry | 1,321.26 |
|  | mD_2_2021_ | Foraging depth | f(Bathymetry, model="rw2") | 1,021.55 |
|  | mD_3_2021_ | Foraging depth | Bathymetry + f(LoggerID, model="iid") | 1,206.18 |
|  | mD_4_2021_ | Foraging depth | Bathymetry + f(spatial, model=spde21_depth) | **882.59** |
|  | mD_5_2021_ | Foraging depth | Bathymetry + f(LoggerID, model="iid") + f(spatial, model=spde21_depth) | 882.55 |
|  | mD_6_2021_ | Foraging depth | f(Bathymetry, model="rw2") + f(LoggerID, model="iid") + f(spatial, model=spde21_depth) | 882.67 |
| 2021 | mT_1_2021_ | Foraging temperature | SST | 762.94 |
|  | mT_2_2021_ | Foraging temperature | f(SST, model="rw2") | 620.12 |
|  | mT_3_2021_ | Foraging temperature | SST + f(LoggerID, model="iid") | 589.90 |
|  | mT_4_2021_ | Foraging temperature | SST + f(spatial, model=spde21_temp) | **240.59** |
|  | mT_5_2021_ | Foraging temperature | SST + f(LoggerID, model="iid") + f(spatial, model=spde21_temp) | 240.51 |
|  | mT_6_2021_ | Foraging temperature | f(SST, model="rw2") + f(LoggerID, model="iid") + f(spatial, model=spde21_temp) | 240.34 |
| 2023 | mD_1_2023_ | Foraging depth | Bathymetry | 1,286.00 |
|  | mD_2_2023_ | Foraging depth | f(Bathymetry, model="rw2") | failed |
|  | mD_3_2023_ | Foraging depth | Bathymetry + f(LoggerID, model="iid") | 1,191.99 |
|  | mD_4_2023_ | Foraging depth | Bathymetry + f(spatial, model=spde23_depth) | **1,084.88** |
|  | mD_5_2023_ | Foraging depth | Bathymetry + f(LoggerID, model="iid") + f(spatial, model=spde23_depth) | 1,081.98 |
|  | mD_6_2023_ | Foraging depth | f(Bathymetry, model="rw2") + f(LoggerID, model="iid") + f(spatial, model=spde23_depth) | failed |
| 2023 | mT_1_2023_ | Foraging temperature | SST | 865.74 |
|  | mT_2_2023_ | Foraging temperature | f(SST, model="rw2") | 427.49 |
|  | mT_3_2023_ | Foraging temperature | SST + f(LoggerID, model="iid") | 780.77 |
|  | mT_4_2023_ | Foraging temperature | SST + f(spatial, model=spde23_temp) | **186.46** |
|  | mT_5_2023_ | Foraging temperature | SST + f(LoggerID, model="iid") + f(spatial, model=spde23_temp) | 186.45 |
|  | mT_6_2023_ | Foraging temperature | f(SST, model="rw2") + f(LoggerID, model="iid") + f(spatial, model=spde23_temp) | 186.41 |

**Table S5.** Generalized linear models (family negative binomial) with a zero-inflation term describing factors affecting *Calanus* spp. abundance in water (based on LOPC-CTD data) with different interaction terms. Models ordered with ascending Akaike Information Criterion (AIC) of particular model with the lowest AIC corresponding to the best model.

| **Model** | **AIC** |
| --- | --- |
| *Calanus* spp. abundance ~ SeaTemp*Year + Depth*Year + SeaTemp:Depth:Year  *Zero inflation: ~Depth + Temperature* | 968,028.9 |
| *Calanus* spp. abundance ~ SeaTemp*Year + Depth*Year + SeaTemp*Depth  *Zero inflation: ~Depth + Temperature* | 974,905 |
| *Calanus* spp. abundance ~ SeaTemp*Year + Depth*Year  *Zero inflation: ~Depth + Temperature* | 976,357 |
| *Calanus* spp. abundance ~ SeaTemp + Depth + Year  *Zero inflation: ~Depth + Temperature* | Convergence problem |

**Table S6.** Summary of the percentages of dives per year in various Foraging Thermal Categories (FTC) and dive type (SSM, IIIU or DLV). All percentages are summing to 100 in rows.

|  | | Dive types | | | | | |
| --- | --- | --- | --- | --- | --- | --- | --- |
|  | | DLV | | SSM | | IIU | |
| Year | FTC | counts | Percentage | counts | Percentage | counts | Percentage |
| 2018 | Cold | 185 | 5.9 | 2,823 | 89.6 | 144 | 4.6 |
|  | Transitional | 79 | 11.8 | 575 | 85.7 | 17 | 2.5 |
|  | Warm | 70 | 43.2 | 90 | 55.6 | 2 | 1.2 |
| 2021 | Cold | 23 | 0.6 | 3,772 | 99.1 | 11 | 0.3 |
|  | Transitional | - | - | 61 | 100 | - | - |
|  | Warm | - | - | 16 | 100 | - | - |
| 2022 | Cold | 4 | 2.2 | 176 | 97.2 | 1 | 0.6 |
|  | Transitional | 2 | 13.3 | 13 | 86.7 | - | - |
|  | Warm | 18 | 32.7 | 34 | 61.8 | 3 | 5.5 |
| 2023 | Cold | 27 | 2.2 | 1,191 | 96.2 | 20 | 1.6 |
|  | Transitional | 57 | 2.3 | 2,397 | 96.9 | 20 | 0.8 |
|  | Warm | 87 | 3.9 | 2,116 | 95.2 | 19 | 0.9 |
| 2024 | Cold | 130 | 2.9 | 4,231 | 96.2 | 36 | 0.8 |
|  | Transitional | 7 | 2.6 | 289 | 95.1 | 8 | 2.3 |
|  | Warm | 2 | 5.0 | 37 | 92.5 | 1 | 2.5 |

**Table S7**. Results of the pairwise *post-hoc* comparison of the estimate temperature slopes from the GLM model at specific depth (5 m, 10 m, and 23 m) among years (2018, 2021, 2022, 2023, and 2024). Bolded p-values are significant. The depths selected are the average depth for each dive type (SSM, IIU, and DLV) for the 5 study years combined.

| *Comparison* | *Depth* | *Estimate* | *SE* | *df* | *z.ratio* | *p.value* |
| --- | --- | --- | --- | --- | --- | --- |
| 2018 - 2021 | 5 | -0.17 | 0.01 | Inf | -16.35 | **<0.0001** |
| 2018 - 2022 |  | -0.12 | 0.01 | Inf | -10.95 | **<0.0001** |
| 2018 - 2023 |  | 0.07 | 0.01 | Inf | 5.23 | **<0.0001** |
| 2018 - 2024 |  | 0.64 | 0.01 | Inf | 67.01 | **<0.0001** |
| 2021 - 2022 |  | 0.06 | 0.01 | Inf | 4.71 | **<0.0001** |
| 2021 - 2023 |  | 0.24 | 0.01 | Inf | 16.60 | **<0.0001** |
| 2021 - 2024 |  | 0.82 | 0.01 | Inf | 70.34 | **<0.0001** |
| 2022 - 2023 |  | 0.18 | 0.01 | Inf | 12.65 | **<0.0001** |
| 2022 - 2024 |  | 0.76 | 0.01 | Inf | 65.90 | **<0.0001** |
| 2023 - 2024 |  | 0.58 | 0.01 | Inf | 41.75 | **<0.0001** |
| 2018 - 2021 | 12 | -0.07 | 0.01 | Inf | -9.05 | **<0.0001** |
| 2018 - 2022 |  | -0.004 | 0.01 | Inf | -0.55 | 0.981 |
| 2018 - 2023 |  | 0.03 | 0.01 | Inf | 3.44 | **0.005** |
| 2018 - 2024 |  | 0.50 | 0.01 | Inf | 69.12 | **<0.0001** |
| 2021 - 2022 |  | 0.07 | 0.01 | Inf | 7.26 | **<0.0001** |
| 2021 - 2023 |  | 0.11 | 0.01 | Inf | 9.82 | **<0.0001** |
| 2021 - 2024 |  | 0.57 | 0.01 | Inf | 64.27 | **<0.0001** |
| 2022 - 2023 |  | 0.04 | 0.01 | Inf | 3.46 | **0.005** |
| 2022 - 2024 |  | 0.50 | 0.01 | Inf | 57.23 | **<0.0001** |
| 2023 - 2024 |  | 0.46 | 0.01 | Inf | 45.64 | **<0.0001** |
| 2018 - 2021 | 26 | 0.14 | 0.13 | 0.01 | Inf | **<0.0001** |
| 2018 - 2022 |  | 0.23 | 0.22 | 0.01 | Inf | **<0.0001** |
| 2018 - 2023 |  | -0.02 | -0.04 | 0.01 | Inf | **0.001** |
| 2018 - 2024 |  | 0.12 | 0.20 | 0.01 | Inf | **<0.0001** |
| 2021 - 2022 |  | 0.09 | 0.09 | 0.01 | Inf | **<0.0001** |
| 2021 - 2023 |  | -0.17 | -0.16 | 0.01 | Inf | **<0.0001** |
| 2021 - 2024 |  | -0.02 | 0.08 | 0.01 | Inf | **<0.0001** |
| 2022 - 2023 |  | -0.25 | -0.25 | 0.01 | Inf | **<0.0001** |
| 2022 - 2024 |  | -0.11 | -0.01 | 0.01 | Inf | 0.285 |
| 2023 - 2024 |  | 0.14 | 0.24 | 0.01 | Inf | **<0.0001** |

**References**

Aho, K. (2025). *asbio: A Collection of Statistical Tools for Biologists*. https://doi.org/10.32614/CRAN.package.asbio

Fleming, C. H., & Calabrese, J. M. (2023). *ctmm: Continuous-Time Movement Modeling*. https://github.com/ctmm-initiative/ctmm

Luque, S. P. (2024). *diveMove: Dive Analysis and Calibration.R package version 1.6.2*. https://cran.r-project.org/package=diveMove

Lysy, M., Stasko, A. D., & Swanson, H. K. (2023). *nicheROVER: Niche Region and Niche Overlap Metrics for Multidimensional Ecological Niches*. <https://cran.r-project.org/package=nicheROVER>

Sullivan GM, Feinn R. Using Effect Size—or Why the P Value Is Not Enough. J Grad Med Educ. 2012;4(3):279–82. doi:10.4300/jgme-d-12-00156.1 PubMed PMID: 23997866.

Vihtakari, M. (2024). *ggOceanMaps: Plot Data on Oceanographic Maps using “ggplot2.”* https://doi.org/10.32614/CRAN.package.ggOceanMaps
